# Cyclic AMP Affects Oocyte Maturation and Embryo Development in Prepubertal and Adult Cattle

**DOI:** 10.1371/journal.pone.0150264

**Published:** 2016-02-29

**Authors:** Sandra Milena Bernal-Ulloa, Julia Heinzmann, Doris Herrmann, Klaus-Gerd Hadeler, Patrick Aldag, Sylke Winkler, Dorit Pache, Ulrich Baulain, Andrea Lucas-Hahn, Heiner Niemann

**Affiliations:** 1 Institute of Farm Animal Genetics, Biotechnology, Friedrich-Loeffler-Institut, Mariensee, Germany; 2 Facultad de Ciencias Agropecuarias, Universidad de Ciencias Aplicadas y Ambientales, Bogotá, Colombia; 3 DNA Sequencing Facility, Max Planck Institute of Molecular Cell Biology and Genetics, Dresden, Germany; Institute of Zoology, Chinese Academy of Sciences, CHINA

## Abstract

High cAMP levels during *in vitro* maturation (IVM) have been related to improved blastocyst yields. Here, we employed the cAMP/cGMP modulators, forskolin, IBMX, and cilostamide, during IVM to unravel the role of high cAMP in early embryonic development produced from prepubertal and adult bovine oocytes. Oocytes were collected via transvaginal aspiration and randomly assigned to three experimental groups: TCM24 (24h IVM/control), cAMP30 (2h pre-IVM (forskolin-IBMX), 30h IVM-cilostamide), and DMSO30 (Dimethyl Sulfoxide/vehicle control). After IVM, oocytes were fertilized *in vitro* and zygotes were cultured *in vitro* to blastocysts. Meiotic progression, cAMP levels, mRNA abundance of selected genes and DNA methylation were evaluated in oocytes. Blastocysts were used for gene expression or DNA methylation analyses. Blastocysts from the cAMP30 groups were transferred to recipients. The cAMP elevation delayed meiotic progression, but developmental rates were not increased. In immature oocytes, mRNA abundance of *PRKACA* was higher for cAMP30 protocol and no differences were found for *PDE3A*, *SMAD2*, *ZAR1*, *PRDX1* and *SLC2A8*. *EGR1* gene was up-regulated in prepubertal cAMP30 immature oocytes and down-regulated in blastocysts from all *in vitro* treatments. A similar gene expression profile was observed for *DNMT3b*, *BCL2L1*, *PRDX1* and *SLC2A8* in blastocysts. Satellite DNA methylation profiles were different between prepubertal and adult oocytes and blastocysts derived from the TCM24 and DMSO30 groups. Blastocysts obtained from prepubertal and adult oocytes in the cAMP30 treatment displayed normal methylation profiles and produced offspring. These data indicate that cAMP regulates IVM in prepubertal and adult oocytes in a similar manner, with impact on the establishment of epigenetic marks and acquisition of full developmental competency.

## Introduction

Cyclic adenosine monophosphate (cAMP) is a second messenger involved in many cellular functions. In mammalian oocytes it maintains meiotic arrest by inactivation of maturation promoting factor (MPF) and by stimulating cAMP-dependent protein kinase A (PKA). During *in vivo* maturation in the mouse model, LH induces a decrease in cyclic guanosine monophosphate (cGMP) in the mural granulosa cells and the oocyte, which in turn relieves the inhibition of phosphodiesterase 3A, by hydrolyzing cAMP to AMP, inducing activation of MPF and ultimately germinal vesicle breakdown (GVBD) [1].

Cyclic AMP controls meiotic progression in fetal ovaries, and is involved in the establishment of the primordial follicle pool [2]. However, the ability to accumulate cAMP is reduced in oocytes from prepubertal pigs [3] and mice [4]. Since the oocyte receives cAMP from the adjacent cumulus cells via gap junctions, the lower cAMP levels associated with lower developmental capacity in prepubertal oocytes have been linked either to decreased LH/FSH ovarian receptor expression [5], altered adenylate cyclase response [4], different phosphodiesterase (PDE) activities [4], and/or defective gap junction communication [6].

When the oocyte is mechanically released from the antral follicle for *in vitro* maturation, intra-oocyte cAMP levels decrease and meiotic resumption begins non-physiologically, via “spontaneous” or “pseudo” maturation, attributed to the removal of inhibitory factors from the follicle rather than active processes [7, 8]. This “pseudo” maturation has been linked to the lower efficiency reported for *in vitro* embryo production systems [9].

Diverse strategies have been tested not only in adult but also in prepubertal females to improve *in vitro* embryo production, including donor ovarian stimulation with different hormones or growth factors, such as eCG [10], GnRH [11], FSH [12], or IGF1 [13, 14] with or without previous progesterone treatment [15, 16]. Furthermore, modulation of oocyte meiotic resumption by cAMP modulator agents [17], MPF inhibition [18], or cAMP analogs [3] have also been suggested. The simulated physiological oocyte maturation (SPOM) method was recently proposed to increase cAMP levels in bovine oocytes and cumulus cells from adult donors. It prevented spontaneous resumption of meiosis after mechanical oocyte retrieval and thereby improved *in vitro* embryo development [19]. SPOM includes two steps using three different cAMP regulator compounds, forskolin (adenylate cyclase stimulator) and 3-isobutyl-1-methylxanthine (IBMX, non-specific inhibitor of phosphodiesterases) 2 h prior to IVM followed by an extended exposure (30 h) to cilostamide (selective inhibitor of PDE3A and PDE3B).

The use of prepubescent oocytes is a promising strategy to preserve fertility in female pediatric patients with malignant disease or Turner syndrome [20, 21]. However, little is known about the competence of these oocytes and ethical reasons prohibit research on humans. Female prepubertal bovine donors have been used for *in vitro* embryo production with controversial results, but are currently used for commercial purposes [14, 22]. The bovine model has been widely used for reproductive studies due to remarkable similarities with human [23]. Nevertheless, to the best of our knowledge no research has been performed evaluating the effects of high cAMP levels on the developmental potential of bovine prepubertal oocytes. The purpose of the present study was to unravel the effects of increased intra-oocyte cAMP levels during maturation in bovine oocytes derived from prepubertal and adult donors using the SPOM system. We investigated the effects of elevated cAMP levels on mRNA expression of developmentally important genes, the CpG DNA methylation profiles of two DNA-satellite sequences and *in vitro* and *in vivo* development of oocytes from prepubertal and adult bovine donors.

## Materials and Methods

Bovine oocytes from prepubertal and adult donors were used in three different *in vitro* maturation (IVM) protocols. *In vivo* matured oocytes and *in vivo* produced expanded blastocysts were used as physiological standards for comparison with their *in vitro* produced counterparts. All bovine female donors and recipients were selected from the experimental herds of the Institute of Farm Animal Genetics in Mariensee (Germany). Experiments were performed according to the German Animal Welfare regulations and had been approved by the local supervisory body (LAVES).

### Donors management for oocyte retrieval

Sixty six Holstein Friesian prepubertal female donors 6–9 mo old (7.4 on average) and 66 adult fertile Holstein Friesian donors, 4.4 years old on average (> 2 lactations, 50–180 d postpartum, 150.4 d average), were employed in groups of 12 (6 adult and 6 prepubertal donors) for transvaginal ultrasound oocyte recovery (ovum pick up, OPU) twice per week at 3–4 days interval. Animals underwent careful examination of the general health status and adequate development and condition of the reproductive organs prior to the experiments. The dominant follicles were removed by OPU 4 days prior to starting with oocyte collection in all treatment groups [14]. Donors were rotated among treatments for every OPU session, to eliminate any donor specific effects in the various experimental groups.

### Ultrasound-guided oocyte retrieval

Transvaginal ultrasound ovum pick up (OPU) was performed as previously reported [14, 16]. Briefly, an Aloka real-time B-mode ultrasound system (Aloka SSD-4000, Hitachi Aloka Medical Ltd., Tokyo, Japan) and a 7.5 MHz electronic convex transducer (Hitachi Aloka Medical Ltd., Tokyo, Japan) were used to visualize the ovaries (S1 Fig). Visible follicles ≥ 3 mm in diameter were punctured using a disposable 20G x 2 ¾” needle (0.9 x 70 mm, Terumo, Eschborn, Germany). A vacuum pump (Aspirator 3, Labotect GmbH, Göttingen, Germany), adjusted to a negative pressure of 60 mmHg (20 ml/min) was used to recover follicular fluid. After puncture of four to five follicles, the oocyte collection system was flushed with Dulbecco’s PBS medium (AppliChem, Darmstadt, Germany), supplemented with 2.2 IU/ml heparin (AppliChem), 1% newborn calf serum (NBCS; PPA Laboratories, Coelbe, Germany), 6 μg/ml penicillin G (AppliChem) and 50 μg/ml streptomycin sulphate (AppliChem) for the standard protocol (TCM24) or additionally supplemented with 3-isobutyl-1-methylxanthine (IBMX, 500 μM, Sigma-Aldrich) for the protocol using the cAMP regulators (cAMP30) and dimethyl sulfoxide (DMSO, 46.6 mM, Sigma-Aldrich) as vehicle control (DMSO30). The vehicle control was necessary because IBMX was dissolved in DMSO. The interval from ovum pick up to oocyte searching did not exceed 20 min.

### Protocols for *in vitro* maturation (IVM)

Standard IVM (TCM24): Collected oocytes were washed in 3 ml of TCM-air medium which consists of Tissue Culture Medium 199 (TCM199, Sigma-Aldrich, St. Louis, MO, USA), enriched with 50 μg/ml gentamicin sulphate (Sigma-Aldrich), 0.2 mM Na- pyruvate (Sigma-Aldrich), 4.2 mM NaHCO3 (Honeywell Riedel-de Haën, Seelze, Germany) and 1 mg/ml BSA-FAF (Sigma-Aldrich). Thereafter, oocytes were placed into drops (50μl) under oil using the same medium. Cumulus-oocyte complexes were classified into five morphological categories [14] and only oocytes from categories I, II and III were used for *in vitro* maturation (IVM): Category I: Oocytes with more than four layers of compact cumulus cells and a homogeneous cytoplasm. Category II: Oocytes with three to four layers of compact cumulus cells, homogeneous cytoplasm or lightly granulated. Category III: Oocytes with one to two layers of cumulus of corona radiata, cytoplasm with irregular appearance, such as dark areas. Category IV: Denuded oocytes. Category V: Oocytes with expanded cumulus (S2 and S3 Figs). For IVM, selected oocytes were washed three times in 100 μl drops of TCM-culture medium, which is contains Tissue Culture Medium 199 (TCM199), 0.2 mM Na-pyruvate (Sigma-Aldrich), 25 mM NaHCO_3_ (Honeywell Riedel-de Haën)_,_ 50 μg/ml gentamycin (Sigma-Aldrich) and 1 mg/ml FAF-BSA (Sigma-Aldrich). Oocytes from each donor were incubated separately in 50 μl TCM-culture drops under silicone oil, supplemented with 10 UI/ml of equine chorion gonadotropin (eCG) and 5 IU/ml of human chorionic gonadotropin (hCG) (Suigonan^®^, Intervet, Unterschleissheim, Germany). Oocytes were incubated in a humidified atmosphere at 39°C and 5% CO_2_ in air for 24h.

Extended IVM system (cAMP30): Oocytes considered viable (morphological categories I, II and III, S2 and S3 Figs) from every donor were washed in 3 ml TCM-air medium, additionally supplemented with 500 μM 3-isobutyl-methilxanthine (IBMX, Sigma-Aldrich) and 100 μM forskolin (FSK, Sigma-Aldrich) (SPOM system) [19] and then maintained prior to IVM (pre-IVM) for 2 h in 50 μl drops of the same medium under silicone oil at 39°C. After the pre-IVM phase, oocytes were washed three times in 100 μl drops of TCM-culture medium under oil, and were matured *in vitro* in 50 μl drops of TCM-culture medium, supplemented with 20 μM cilostamide and Suigonan^®^ as described before. Incubation was performed in a humidified atmosphere at 39°C, 5% CO_2_ in air for 30 h.

Vehicle control (Dimethyl Sulfoxide, DMSO30): DMSO was used as solvent for all cAMP modulators for the cAMP30 treatment. Therefore, it was used at the same concentrations and time periods as vehicle control. Viable oocytes from each donor (categories I, II and III, S2 and S3 Figs) were washed in 3 ml of TCM-air medium, supplemented with 280 mM (2%) DMSO and after cultured for 2 h prior to IVM at 39°C in 50 μl drops under silicone oil using the same medium. After pre-IVM culture, oocytes were washed three times in 100 μl drops of TCM-culture medium, and matured *in vitro* in 50 μl drops of TCM-culture medium under silicone oil, supplemented with 5.6 mM (0.04%) DMSO and Suigonan^®^. *In vitro* maturation was carried out in a humidified atmosphere at 39°C, 5% CO_2_ in air for 30 h.

### Monitoring progression through meiosis

Cumulus cells were completely removed from subsets of oocytes from prepubertal and adult donors after 9, 20 and 24 or 30 h after onset of IVM, according to each protocol, by 5 min. incubation in phosphate buffered saline (PBS), supplemented with 0.1% hyaluronidase (Sigma-Aldrich) and 1mg/ml BSA (Fraction V, Sigma-Aldrich) at 38° followed by vortexing for 5 min at 1400 rpm. Denuded oocytes were fixed in a 2% PBS-glutaraldehyde solution and maintained at 4°C until evaluation. After fixation, oocytes were stained with Hoechst 33258 (0.01 mg/ml, Sigma-Aldrich) for ten minutes. To evaluate the nuclear status, oocytes were evaluated under a fluorescence microscope. The number and proportion of oocytes either in germinal vesicle (GV), germinal vesicle breakdown (GVBD), metaphase I (MI), or metaphase II (MII) stages and non-evaluable (NE) were recorded (S4 Fig). A total of 614 oocytes from prepubertal donors and 632 oocytes from adult females were fixed and analyzed in four replicates. Data are expressed in percentages calculated on the total number of oocytes per treatment per fixation time.

### Collection of immature and *in vitro* matured oocytes

Immature oocytes from prepubertal and adult donors after either OPU (TCM24 protocol) or after 2h pre-IVM culture (DMSO30 and cAMP30) were denuded as mentioned above. Denuded immature and *in vitro* matured oocytes in MII, indicated by the presence of the first polar body, were frozen at -80°C, individually or in groups of five in PBS supplemented with 0.1% polyvinyl alcohol (PVA, Sigma-Aldrich) (PBS-PVA solution) for further analysis.

### Measurement of cAMP levels in denuded oocytes

Cyclic AMP levels were determined in immature and *in vitro* matured oocytes from prepubertal and adult donors (categories I, II, III) using a cAMP ELISA test kit (96 Well Enzyme-linked Immunosorbent Assay Kit, Enzo Life Science, NY, USA), according to manufacturer’s instructions. Immature oocytes either after OPU (TCM24 protocol) or 2 h pre-IVM culture (DMSO30 and cAMP30 protocols) and matured oocytes from all protocols (24 or 30 h IVM) were denuded and incubated in 0.1M HCl for 10 min at room temperature. After lysis, centrifugation was performed at 14.000 rpm for 15 min at 4°C. The supernatant was stored frozen at -20°C until ELISA analysis was performed. Immediately prior to the assay, all standards and samples were acetylated according to manufacturer instructions. Optical density at 405 nm was measured in a Tecan Sunrise ™ 96-well microplate absorbance ELISA- reader (Tecan Group Ltd., Männedorf, Switzerland). A total of 1800 denuded immature (900) and MII (900) oocytes were employed for this study. Three pools of 50 immature and 50 MII denuded oocytes each, from all treatment groups (cAMP30, DMSO30, TCM24), from both prepubertal and adult donors, were analyzed.

### *In vitro* fertilization (IVF) and *in vitro* embryo development (IVC)

Matured cumulus-oocyte complexes from each group age and IVM treatment were washed three times in fertilization medium (Fert-TALP)[24], containing 6 mg/ml BSA (fraction V, Sigma-Aldrich), 0.05 mg/ml gentamicin (Sigma-Aldrich) and 0.028 mg/ml Na-pyruvate (Sigma-Aldrich) and transferred into 100 μl drops under silicone oil of 100 μl of Fert-TALP enriched with 10 μM hypotaurine (Sigma-Aldrich), 0.1 IU/ml heparin (AppliChem), and 1 μM epinephrine (Sigma-Aldrich). Frozen/thawed sperm from one bull of proven fertility was used throughout these experiments. Two gradients of Bovipure™ (Nidacon, Gothenburg, Sweden), 40% and 80%, respectively, were prepared to obtain motile spermatozoa after centrifugation at 300 g for 10 min. Spermatozoa were added to reach a final concentration of 1 × 10^6^ cells/ ml and were co-incubated with oocytes for 19 h, in a humidified atmosphere of 5% CO_2_ in air at 39°C. After fertilization, presumptive zygotes from all protocols were denuded by vortexing for 1 min in TCM-air medium. All denuded zygotes were washed three times in synthetic oviductal fluid (SOF) medium enriched with 4 mg/ml of BSA-FAF (Sigma-Aldrich) and groups of five zygotes were cultured in 30 μl droplets of SOF under silicone oil at 39°C, 5% CO_2_ and 5% O_2_. Cleavage rates and blastocyst formation were evaluated 48 h and 8 d after IVF respectively. Expanded blastocysts on day 8 were individually frozen and stored at –80°C in PBS-PVA solution until further use.

### Production of *in vivo* matured oocytes

*In vivo* matured oocytes were produced as “physiological” controls for gene expression and DNA methylation studies. Cycling adult Holstein Friesian female donors were selected for collecting *in vivo* matured oocytes after hormonal stimulation. Ten to eleven days after natural estrus, the dominant follicle was removed by ovum pick up (OPU). Two days later, decreasing doses of FSH/LH from porcine pituitary extract (Pluset®, Calier S.A., Barcelona, Spain) were injected intramuscularly (IM) twice per day (12 h interval) for 4 d as follows: day 1, 100 IU FSH; day 2, 75 IU FSH; day 3, 50 IU FSH; day 4, 25 IU FSH. A total of 500 μg of the PGF2α analogue cloprostenol (Estrumate®, Intervet, Unterschleissheim, Germany) was IM injected along with the second dose of FSH of day 3 and the first FSH dose of day 4. Sixteen hours after the last dose of FSH, 12 μg buserelin (Receptal®, Intervet, Unterschleissheim, Germany) were intravenously injected. Oocytes were retrieved by ovum pick up 24 h after buserelin administration. Cumulus cells were removed (see progression through meiosis procedure) and metaphase II oocytes were determined by the presence of the first polar body. Denuded MII oocytes were frozen individually or in groups of five in PBS-PVA solution at –80°C until analysis.

### *In vivo* embryo production

Adult Holstein Friesian females were superovulated using a single dose of 2500–3000 UI eCG (Intergonan^®^; Intervet, Tönisvorst, Germany). A single dose of 750 μg cloprostenol (Estrumate^®^) was administered 48 h after eCG. Two artificial inseminations were performed with frozen/thawed sperm from the same bull used for IVF. Embryos were collected non-surgically from the uterine horns on days 7 or 8 as described previously [25]. Dulbecco’s PBS medium (AppliChem), supplemented with 1% fetal calf serum (FCS, Invitrogen, Karlsruhe, Germany), was used for uterine flushing. Recovered expanded blastocysts were frozen individually in a small volume (2–4 μl) of PBS-PVA solution for further analyses.

### *In vivo* development after embryo transfer

*In vivo* development of embryos produced from oocytes matured in the presence of cAMP regulators was assessed to clarify whether or not the extended and modulated *in vitro* maturation period affected the ability of the embryos to establish and maintain pregnancy. Due to the limited number of available recipients, and taking into account previous reports as reference for pregnancy and calving rates obtained under similar *in vitro* conditions for prepubertal and adult oocytes [26], we did not transfer embryos produced with the TCM24 and DMSO30 protocols. Up-to-now, no *in vivo* data had been reported after use of SPOM in the bovine species. Single fresh blastocysts produced from prepubertal or adult oocytes treated with the cAMP30 protocol were non-surgically transferred (ET) to healthy Holstein cycling heifers (18–24 months old), synchronized with the intra-vaginally placed CIDR® (Progesterone 1.38 g, Zoetis, Berlin, Germany) over 7 days. On the time of CIDR removal, one injection of 500 μg of cloprostenol (Estrumate®) was administered followed by a second dose 12 h later. Pregnancies were confirmed by transrectal ultrasound examination or rectal palpation 45, 90 and 180 days after transfer. Calving events and birthweight were recorded at parturition.

### Relative mRNA abundance determination

Transcript profiles of a panel of developmentally important genes were determined in single denuded immature and matured (MII) oocytes and expanded day 8 blastocysts using semi-quantitative reverse transcription real-time polymerase chain reaction (RT-qPCR). The analyzed genes included phosphodiesterase 3A (*PDE3A*), protein kinase cAMP-activated catalytic subunit alpha (*PRKACA*), SMAD family member 2 (*SMAD2*, oocyte aging marker), zygote arrest 1 (*ZAR1*, oocyte-embryo transition), peroxiredoxin 1 (*PRDX1*, antioxidant), solute carrier family 2, member 8 (facilitated glucose transporter, *SLC2A8*) and early growth response protein 1 (*EGR1*, zinc-finger transcription factor). Additionally, in expanded blastocysts, we studied mRNA expression of DNA cytosine-5 methyltransferase 3b (*DNMT3b*, *de novo* methylation), BCL2-like 1(*BCL2L1*, anti-apoptotic regulator), *PRDX1*, *SLC2A8*, and *EGR1*. These genes were selected for this study taking into account their critical role in the acquisition of oocyte developmental competency and early embryo development as reported previously [14, 16, 27]. Samples from every treatment were submitted to poly(A)^+^ mRNA extraction using the Dynabeads^®^ mRNA DIRECT™ KIT (Invitrogen). Cell lysis was achieved using 40 μl of lysis binding buffer (100mM Tris–HCl pH 8.0, 500mM LiCl, 10mM EDTA, 1% lithium dodecyl sulfate, 5mM dithiothreitol) and incubation for 10 min at room temperature. A total of 1pg rabbit globin mRNA (BRL, Gaithersburg, MD, USA) was added as external standard [28] and 5 μl of prewashed Dynabeads^®^ Oligo(dT)_25_ were added to every sample. Incubation was carried out for 15 min at room temperature to bind the poly(A)^+^RNAs to the beads magnetically. Washing was done according to the manufacturer’s instructions. Dynabeads-poly(A)^+^RNAs complexes were resuspended in 11μl water and heated at 68°C for 2.5 min to release the poly(A)^+^ mRNAs, which were immediately used for reverse transcription. Each reverse transcription reaction was performed in a total volume of 20 μl, containing 2 μl of 10X reaction buffer (Life technologies, Carlsbad, USA), 2 μl of dNTPs solution (Bioline Ltd, London, UK), 1 μl of random hexamer primers (Life Technologies), 20 U ribonuclease inhibitor RNAsin^®^ (Life Technologies), 50 U Murine Leukemia Virus Reverse Transcriptase (MuLV, Life Technologies), the extracted poly(A)^+^RNAs and water up to 20 μl. Reverse transcription reactions were performed for 10 min at 25°C, 60 min at 42°C, and 5 min at 99°C.

Semi-quantitative real-time PCR (qPCR) was performed using the obtained cDNA. Reactions were performed in 96-well optical reaction plates (Life Technologies). A final volume of 20 μl, was used per reaction containing 10 μl of 2x Power SYBR^®^Green PCR Master Mix (Life Technologies), 0.8 μl of 5 μM forward and reverse specific primers, 6.4 μl water and 2 μl cDNA. Primer sequences are shown in S1 Table. The mRNA from pools of immature oocytes and expanded blastocysts obtained from slaughterhouse material were extracted and submitted to reverse transcription as described above to create standard curves. A standard curve was performed for each evaluated gene to assess the relative amount of the target gene in each sample. Normalization was performed using the signal from the exogenous standard (rabbit globin) for each sample. RT-qPCR reactions were run for 10 min at 95°C followed by 40 cycles of 15 sec at 95°C, 60 sec at 60°C, 15 sec at 95°C and 1 min at 60°C followed by a slow heating cycle to obtain the dissociation curves. Quantification was performed using the Sequence Detection Software 1.4. A total of 12 replicates were performed per gene.

### DNA methylation profiles assay

The CpG methylation status of the Bovine testis satellite I (BTS) and *Bos taurus* alpha satellite I (BTαS) sequences was determined in denuded immature and matured oocytes and expanded blastocysts obtained from prepubertal and adult female donors using the three different protocols (TCM24, DMSO30, cAMP30). Twelve highly conserved CpG sites were evaluated in a 211 bp fragment for BTS satellite. A fragment of 154 bp including nine CpG sites was analyzed for BTαS [16, 29]. These two DNA satellite sequences have previously been shown to gain insight into CpG site methylation profiles related to the embryo production method in bovine oocytes and embryos [16, 29]. Five single expanded *in vivo* and *in vitro* produced blastocysts, three pools of five immature oocytes either after OPU (TCM24) or 2 h pre-IVM culture (DMSO30, cAMP30), and three pools of five MII oocytes *in vitro* (TCM24, DMSO30 and cAMP30) or *in vivo* derived oocytes were subjected to bisulfite conversion using the EZ DNA Methylation-Direct™ Kit (Zymo Research, Freiburg, Germany). Samples were digested with 13 μl Digestion Buffer, 1 μl Proteinase K, and 12 μl H_2_O at 50°C for 20 min and then centrifuged for 5 min at 10000 x g. Digested samples were submitted to bisulfite conversion using the CT Conversion Reagent provided by the kit at 98°C for 8 min followed by treatment in a thermal cycler at 64°C for 3.5 h. After bisulfite conversion, DNA was washed and cleaned using the Zymo-Spin™ IC Column. Cleaned converted DNA was eluted in 10 μl M-elution buffer. PCR amplification was performed using satellite specific primers (S2 Table) [29]. The correct fragment size was checked on 2% agarose gel. PCR products were purified using the Invisorb® Fragment Cleanup system (Stratec Molecular GmbH, Berlin, Germany). Fragments were ligated into the pGEM^®^-T-Easy Vector (Promega) according to manufacturer instructions overnight at 4°C. Transformation was performed using *Escherichia coli* XL-10 Gold ultracompetent cells (Stratagene, Santa Clara, CA, USA). Screening of positive clones was carried out by direct colony PCR, using SP6 and T7 universal primers (S2 Table). Sequencing was performed using the same set of primers. The BiQ Analyzer program (MPI for Informatics, Saarland, Germany) [30] was used for sequence processing. Sequences from all clones were compared to each specific satellite sequence of the bovine genome. Sequences with a conversion rate lower than 90% or with a high number of sequencing errors in the alignment were excluded from the analysis. Satellite methylation profiles were calculated counting the total methylated CpG sites of the total number of analyzed CpG per treatment.

### Blastocyst cell number determination

A total of six *in vitro* expanded blastocysts (TCM24, DMSO30, cAMP30) obtained from prepubertal and adult donors and *in vivo* expanded blastocysts were submitted to differential staining to identify the total number of cells and the proportion of nuclei in the inner cell mass cells (ICM) and trophectoderm cells (TE) [31]. Day 8 expanded blastocysts were stained with 0.2 mg/ml propidium iodide (Life technologies) for 30 seconds. Embryos were immediately placed and cultured for 4 min in a PBS-PVA solution containing bisBenzimide 0.058 mg/ml (Hoechst 33258, Sigma-Aldrich) and 3.76% formaldehyde (Honeywell Riedel de Haën, Seelze, Germany). Stained blastocysts were mounted on a glycerol drop (Carl Roth GmbH., Karlsruhe, Germany) and observed by fluorescence microscopy. The trophectoderm and the inner cell mass nuclei were identified by the presence of red and blue colors, respectively.

### Statistical analysis

Data from meiotic progression were compared by Fisher´s Exact Test complemented by Bonferroni correction (*P* < 0.016) from R [32]. Two-way ANOVA was used to analyze follicle number, total oocytes and IVM-suitable oocytes per donor per OPU session and cAMP levels in immature and mature oocytes [R or SAS SAS/STAT® software version 9.2 [33]]. Two-way ANOVA followed by Tukey's range test was used to evaluate differences in gene expression (R software). Cleavage and blastocyst rates were compared using the Glimmix procedure from SAS/STAT® software version 9.2 [33]. Methylation profiles were calculated using Chi squared test, followed by pairwise comparison of proportions method from R. Fisher´s extract test was used to evaluate pregnancy rates at days 45, 90 and 180 from R. Due to the low number of observations, blastocysts cell numbers and birth weight are presented descriptively. Except for progression of the meiosis, statistical significance was set at *P* < 0.05. Data are presented as mean ± SEM.

## Results

### Oocyte retrieval in prepubertal and adult donors

A total of 4885 follicles, 4473 retrieved oocytes and 2617 IVM suitable oocytes were recorded for prepubertal donors. For adult donors, 4439 follicles, 3950 retrieved oocytes and 2817 IVM suitable oocytes were obtained. The total number of follicles, oocytes and IVM-suitable oocytes per donor per OPU session were similar among treatment groups and age categories of donors (*P* > 0.05, Table 1).

**Table 1:** Total number of OPU sessions, total number of follicles, total number of oocytes per donor and suitable for IVM obtained via ovum pick up in adult and prepubertal donors.

Donors	Treatment	OPU sessions (n)	Total no. punctured follicles	Total no. retrieved oocytes	Total IVM oocytes	Follicles/Donor*	Obtained oocytes/ Donor*	Oocytes IVM/ Donor*
Prepubertal	cAMP30	216	1944	1744	1040	9.0±0.5	8.1±0.6	4.8±0.4
	DMSO30	198	1627	1461	806	8.2±0.4	7.4±0.5	4.1±0.3
	TCM24	171	1314	1268	771	7.7±0.5	7.4±0.5	4.5±0.3
Adult	cAMP30	202	1691	1527	1099	8.4±0.3	7.6±0.3	5.4±0.2
	DMSO30	180	1461	1294	923	8.1±0.4	7.2±0.3	5.1±0.3
	TCM24	171	1287	1129	795	7.5±0.3	6.6±0.3	4.6±0.3

*Data are the mean ± SEM.

A similar number of follicles and oocytes were observed among treatments and types of donor (*P* > 0.05).

### Meiotic progress

**Fig 1:**
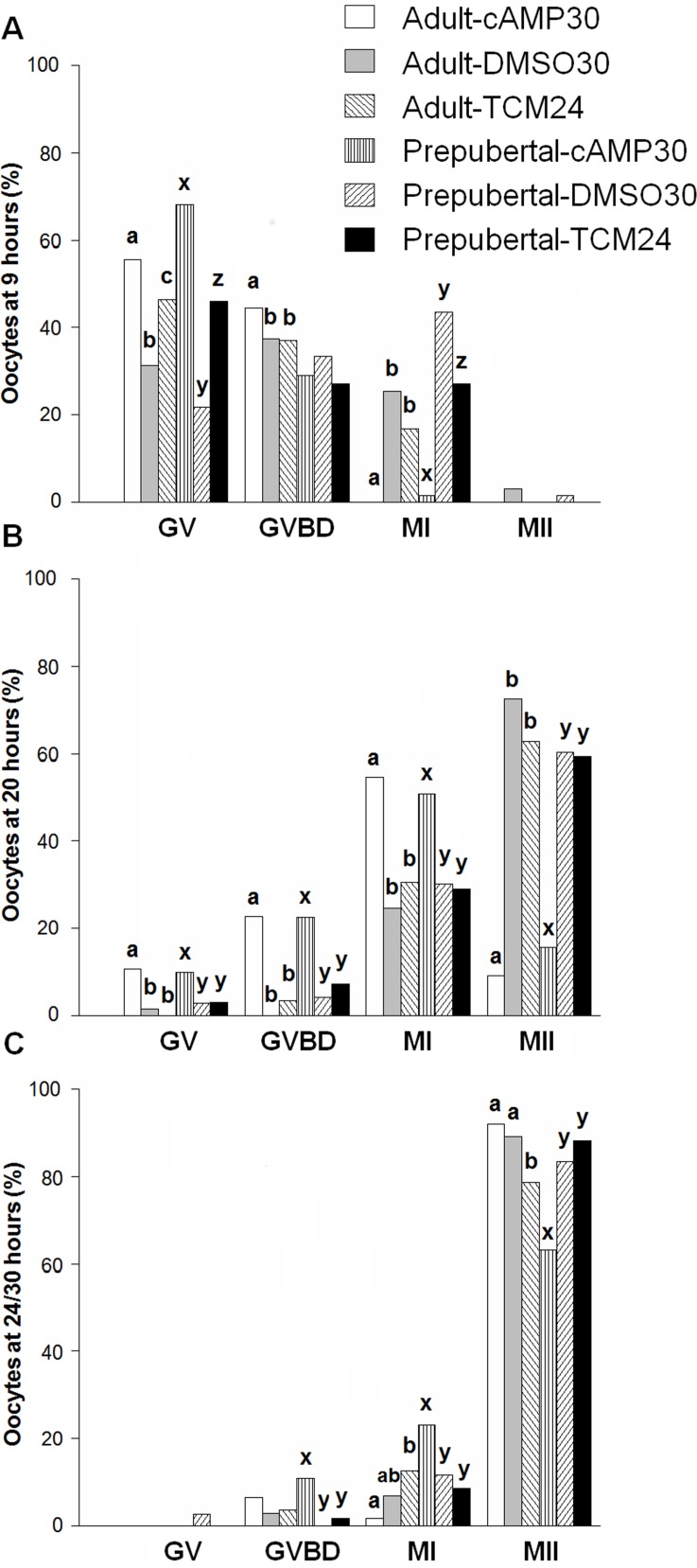
Progression through meiosis of oocytes derived from adult and prepubertal donors collected via OPU and treated prior to and during IVM with or without cAMP modulators. Oocytes were fixed after: A) 9h, B) 20h, and C) 24/30h of *in vitro* maturation. Bars represent the percentages calculated using the total number of oocytes per treatment per fixation time from four replicates. Columns with different superscripts differ significantly among treatments within the group with the respective meiotic status for oocytes obtained from adult (a, b, c) or prepubertal donors (x, y, z). Data were compared using absolute numbers by Fisher´s Exact Test complemented by Bonferroni correction. The percentages were calculated using the total number of samples per treatment and statistical analyses were performed with the absolute values. Therefore, no averages or SEMs are presented. The cAMP modulators delayed progression of meiosis in adult and prepubertal oocytes; DMSO used as solvent for cAMP modulators (vehicle control) accelerated meiotic resumption in oocytes from both types of donors (*P* < 0.016). GV, germinal vesicle stage; GVBD, germinal vesicle breakdown; MI, metaphase I; MII, metaphase II.

At 9 h, cAMP regulators maintained the majority of oocytes from prepubertal and adult donors (GV: 68.1% and 55.5%, respectively) in meiotic arrest compared to standard IVM (GV: 46.1% and 46.3%) and DMSO30 vehicle controls (GV: 21.7% and 31.3%). After 20 h of *in vitro* maturation, the percentage of oocytes that reached MII stage was significantly lower in the cAMP30 protocol, in prepubertal (15.5%) and adult oocytes (9.1%), which was in contrast to the DMSO30 (60.3% and 72.5%, respectively) and TCM24 (59.4% and 62.7%, respectively) groups. After 30 h *in vitro* maturation in the presence of cAMP modulators, metaphase II rates were lower for prepubertal oocytes (63.1%) compared to DMSO30 (83.3%) and TCM24 (88.1%). For adult oocytes, lower MII oocyte rates were observed when standard IVM (78.6%) was performed compared with oocytes treated with cAMP modulators (91.9%) (*P* < 0.016, Fig 1). The detailed data of this experiment are provided in S3 Table.

### Cyclic AMP levels before and after IVM

**Fig 2:**
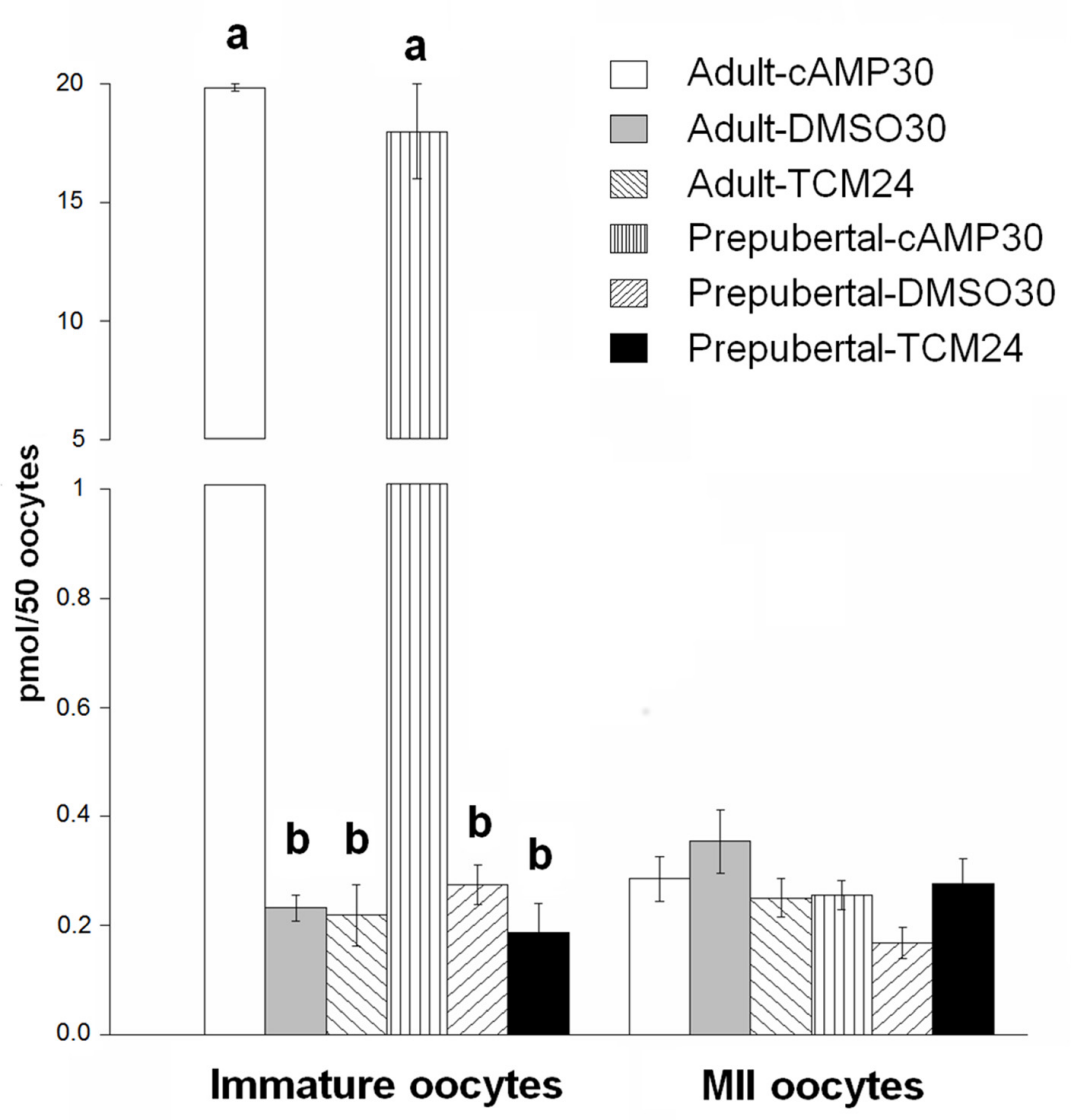
Cyclic AMP levels in prepubertal and adult oocytes treated with or without cAMP modulators prior to and during IVM. Data are presented as mean ± SEM (n = 3). Optical density reads from ELISA test were analyzed using two-way ANOVA. Bars labeled with different superscripts represent statistical significance among treatments (a, b); *P* < 0.05. Oocytes were retrieved via OPU. The cAMP modulators increased intra-oocyte cAMP levels in both prepubertal and adult oocytes after pre-IVM (*P* < 0.05). No differences in cAMP profiles were found among treatments after IVM (*P* > 0.05).

The pre-IVM treatment with IBMX and forskolin (cAMP30 protocol) increased cAMP levels in immature oocytes significantly in both, prepubertal oocytes (18.0±1.98 pmol/50 oocytes) and adult oocytes (19.8±0.15 pmol/50 oocytes) compared with the standard controls (prepubertal: 0.1±0.05 pmol/50 oocytes and adult: 0.2±0.06 pmol/50 oocytes, TCM24), and vehicle controls (prepubertal: 0.2±0.04 and adult: 0.2±0.02, DMSO30) (Fig 2, *P* < 0.05). After 24 or 30 h *in vitro* maturation, cAMP levels were similar among all treatments groups for both prepubertal and adult oocytes (Fig 2, *P* > 0.05).

### Effects of pre-IVM and *in vitro* maturation on *in vitro* embryo development

Cleavage rates did not differ among IVM protocols 48 h after *in vitro* fertilization (*P* > 0.05). The proportion of blastocysts/cultured oocytes was lower in prepubertal oocytes in the vehicle control group (treated with DMSO) than for adult oocytes in the standard (TCM24) and cAMP modulators (cAMP30) treatments, but was similar to the other age and treatment groups (*P* < 0.05, Table 2).

**Table 2:** *In vitro* and *in vivo* developmental rates of prepubertal and adult oocytes cultured pre- and during IVM with or without cAMP modulators.

Donors	Treatment	Replicates (n)	Total retrieved oocytes	Total IVM oocytes	Cleavage rates/IVM oocytes (%)*	Blastocysts rates/IVM oocytes (%)*	Embryo transfer (n)	Pregnancy at 45 d, n(%)	Pregnancy at 90 d, n(%)	Pregnancy at 180 d, n(%)
Prepubertal	cAMP30	18	352	217	57.1±5.2	18.3±2.8 ^ab^	17	7(41.2)	6(35.3)	5(29.4)
	DMSO30	18	406	222	49.5±3.3	13.7±2.0^b^	——	——	——	——
	TCM24	18	306	208	62.0±3.6	23.3±5.1 ^ab^	——	——	——	——
Adult	cAMP30	18	257	162	58.8±5.4	26.5±3.0^a^	15	9(60)	7(46.7)	7(46.7)
	DMSO30	18	271	190	51.1±4.2	20.3±2.9^ab^	——	——	——	——
	TCM24	18	257	206	53.0±5.5	25.9±3.3^a^	——	——	——	——

* Data are the mean ± SEM.

Oocyte retrieval was performed via OPU. Different superscripts indicate statistical significances among treatments groups (a, b); *P* < 0.05. Lower blastocyst yields were found for oocytes treated with the DMSO30 protocol. Similar pregnancy rates were observed for blastocysts produced from prepubertal and adult donors using the cAMP30 treatment.

### Pre-IVM and IVM affect relative mRNA abundance in immature and matured oocytes

**Fig 3:**
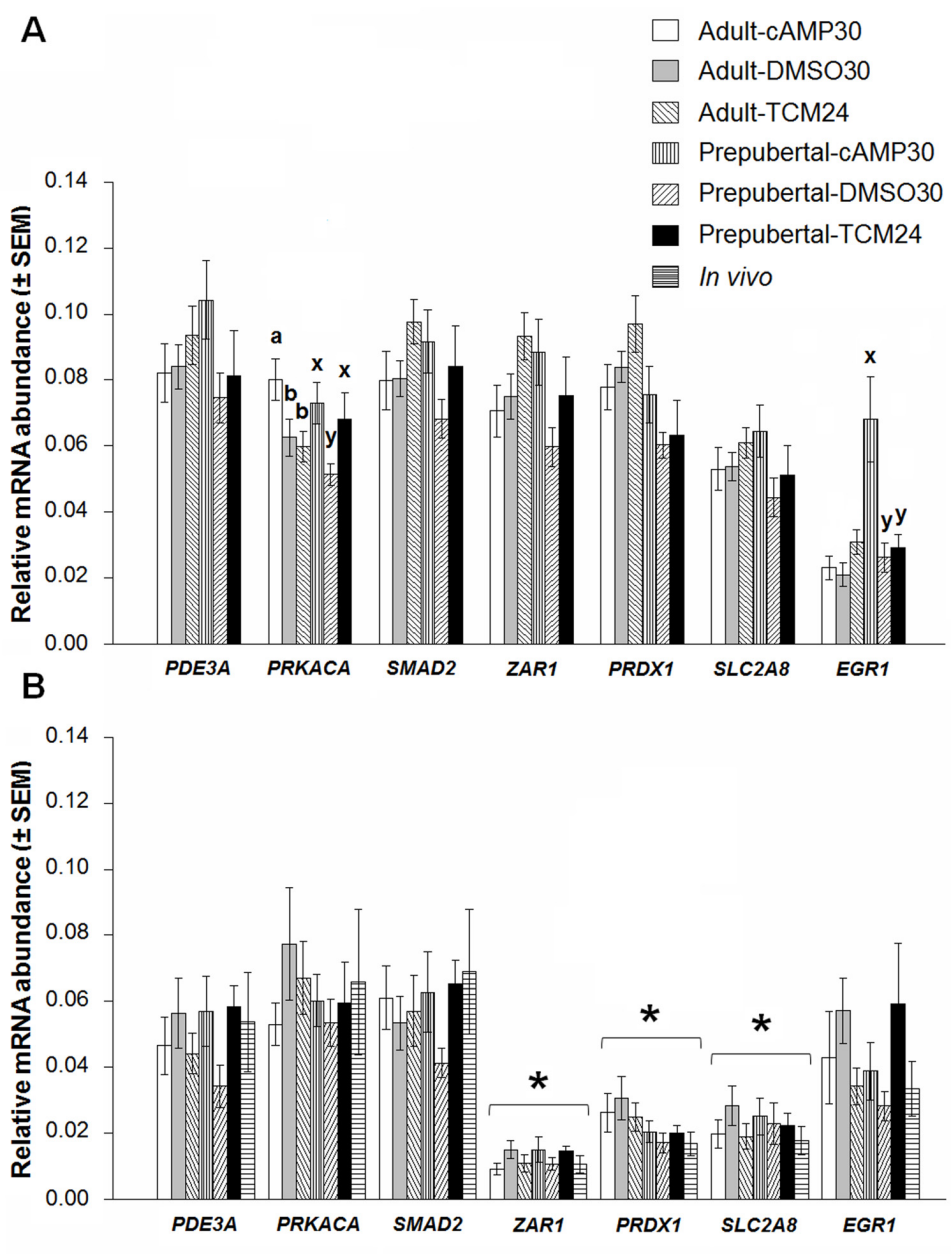
Gene expression profiles in adult and prepubertal oocytes treated with or without cAMP modulators prior to and during IVM. A) single immature oocytes and B) single MII oocytes. Data are presented as mean ± SEM (n = 12). Data were analyzed using two-way ANOVA followed by a Tukey's range test. Columns with different superscripts differ significantly among treatments within the respective meiotic status for oocytes obtained from adult (a, b) and prepubertal donors (x, y). The asterisk represents a statistically significant difference between immature and matured oocyte treatments for the same transcript; *P* < 0.05. Oocytes were obtained via OPU. *In vivo* matured oocytes were used for comparison. *PRKACA* was upregulated in adult oocytes treated with cAMP modulators and down-regulated in prepubertal oocytes under the DMSO30 treatment. *EGR1* was upregulated in prepubertal immature oocytes. Matured oocytes from all treatments displayed lower transcript levels for *ZAR1*, *PRDX1* and *SLC2A8* after IVM compared to immature oocytes.

Immature oocytes from adult donors in the presence of cAMP modulators showed up-regulation of protein kinase cAMP-activated catalytic subunit alpha (*PRKACA*) compared with the control adult group (Fig 3A, *P* < 0.05). The same gene was down-regulated in prepubertal immature oocytes in the DMSO vehicle control (DMSO30) compared to prepubertal control oocytes (TCM24). The relative mRNA abundance of early growth response protein 1 (*EGR1*) gene was significantly up-regulated in prepubertal immature oocytes in the presence of cAMP modulators during OPU and 2 h pre-IVM (Fig 3A, *P* < 0.05). No significant changes in the relative mRNA abundance were detected for *PDE3A*, *SMAD2*, *ZAR1*, *PRDX1*, and *SLC2A8* in immature oocytes across all treatments for both types of donors (Fig 3A, *P* > 0.05).

In MII oocytes, the relative abundances of the selected genes were similar among treatments and donor groups compared with their *in vivo* matured counterparts (Fig 3B, *P* > 0.05). The comparison between immature and MII oocytes revealed significant reduction in transcript levels for *ZAR1*, *PRDX1* and *SLC2A8* in MII oocytes for all *in vitro* treatments (Fig 3B, *P* < 0.05).

### *In vitro* maturation affects relative abundance of selected genes in expanded blastocysts

**Fig 4:**
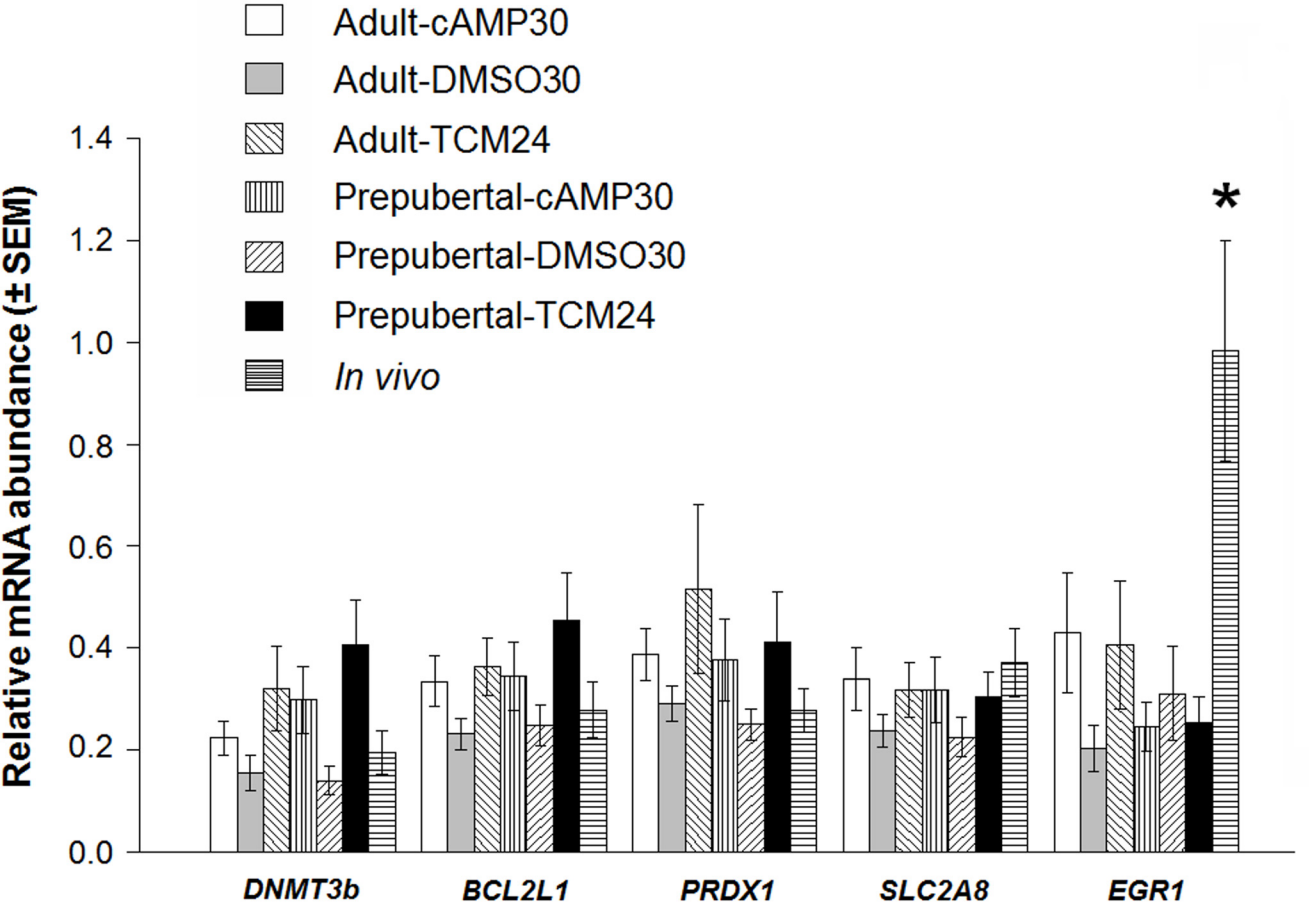
Gene expression profiles in expanded blastocysts produced from adult and prepubertal oocytes treated with or without cAMP modulators prior to and during IVM. Data are shown as the mean ± SEM (n = 12). Data were analyzed using two-way ANOVA followed by a Tukey's range test. The asterisk represents statistical significance among treatments for the same transcript; *P* < 0.05. *In vivo* produced expanded blastocysts were used for comparison. The mRNA relative abundance of the EGR1 gene was lower in all *in vitro* derived blastocysts compared to *in vivo* produced counterparts. No differences among treatments were found for *DNMT3b*, *BCL2L1*, *PRDX1*, *SLC2A8*.

The relative abundance of Early growth response protein 1 (*EGR1*) was significantly reduced in all *in vitro* expanded blastocysts irrespective of treatment (TCM24, cAMP30 and DMSO30) and age of the donors compared to the *in vivo* controls (Fig 4, *P* < 0.05). Similar mRNA abundances were observed among the different *in vitro* protocols for *DNMT3b*, *BCL2L1*, *PRDX1* and *SLC2A8* genes in expanded blastocysts produced *in vitro* or *in vivo* (Fig 4, *P* > 0.05).

### Oocyte and blastocyst DNA methylation levels are affected by cAMP regulators

**Fig 5:**
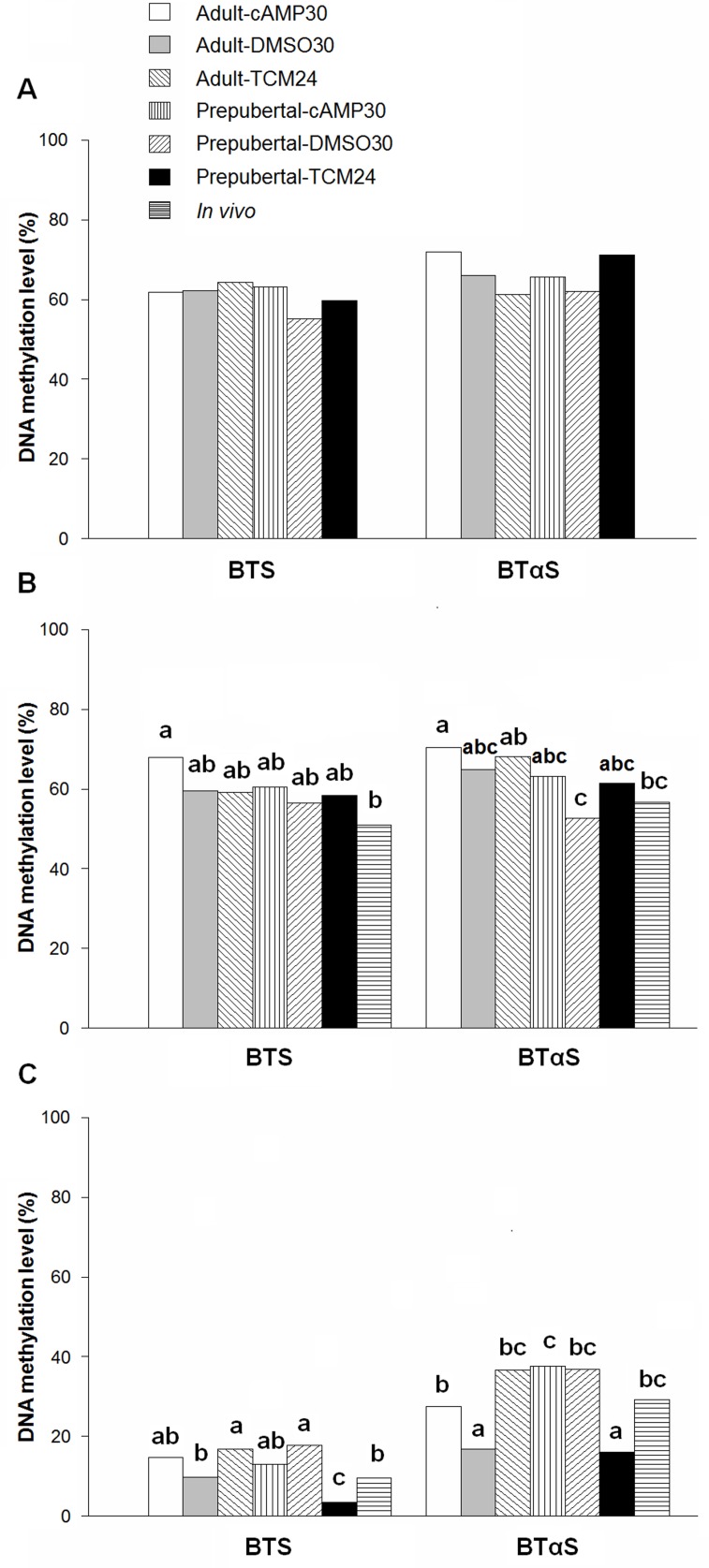
DNA satellite methylation profiles in immature oocytes, MII oocytes and expanded blastocysts derived from adult and prepubertal donors. Oocytes were obtained via OPU and treated prior to and during IVM with cAMP modulators. A) Immature oocytes, B) Matured oocytes and C) Expanded blastocysts. The percentages were calculated using the total number of samples per treatment and statistical analyses were performed with the absolute values. Therefore, no averages or SEMs are presented. Bars represent the percentages of DNA methylation calculated using the total number of CpG analyzed in each treatment. Columns with different superscripts differ significantly for the respective satellite and developmental status (a, b, c). Data were analyzed by Chi-squared test followed by pairwise comparison of proportions method; immature and MII oocytes, n = 3; blastocysts, n = 5; *P* < 0.05. DNA methylation profiles were similar in immature oocytes from all treatments. Hypermethylation was observed for adult matured oocytes treated with cAMP modulators compared with *in vivo* matured oocytes. Lower DNA methylation levels were found for prepubertal oocytes after DMSO treatment compared to adult *in vitro* matured oocytes. Similar DNA methylation levels of both satellite sequences were observed for blastocysts produced from oocytes treated with cAMP modulators and *in vivo* produced blastocysts from both types of donors. DNA methylation levels were aberrant for blastocysts from TCM24 and DMSO30 treatments compared to *in vivo* counterparts. BTS, Bovine testis satellite I; BTαS, *Bos taurus* alpha satellite I.

The methylation patterns of the two satellite sequences did not differ between oocytes derived from prepubertal and adult females after 2 h culture in the presence or absence of cAMP modulators (cAMP30 and DMSO30) or after standard OPU retrieval (TCM24) (immature oocytes, Fig 5A, *P* > 0.05). Significant hypomethylation was observed for BTαS in MII oocytes in vehicle controls derived from prepubertal donors, compared to all *in vitro* treatments of oocytes from adult donors (Fig 5B, *P* < 0.05). After 30 h *in vitro* maturation in the presence of cAMP modulators, oocytes retrieved from adult animals displayed hypermethylation for BTS and BTαS when compared with *in vivo* matured oocytes (Fig 5B, *P* < 0.05). Expanded blastocysts produced from oocytes derived from prepubertal and adult females matured in the presence of cAMP modulators exhibited methylation patterns in both BTS and BTαS satellites similar to *in vivo* produced embryos. The 2 h pre-IVM and 30 h IVM in the vehicle control group or the standard protocol for IVM were associated with aberrant methylation profiles (hyper- or hypomethylation) in embryos obtained from prepubertal or adult donors either in BTS or BTαS sequences in comparison with *in vivo* derived blastocysts (Fig 5C, *P* < 0.05). The number of clones, the number of CpGs analyzed and the mean percentages of methylated CpGs for each protocol and DNA satellite sequences for immature and MII oocytes and expanded blastocysts are shown in S4 Table.

### Cell numbers in expanded blastocysts from the various experimental groups

Prior to staining all blastocysts had a similar morphology. For *in vivo* produced blastocysts the average total number of cells was 155.3±6.8 and for the inner cell mass (ICM) 44.3±4.8. For the *in vitro* produced blastocysts the total cell number oscillated between 134.0±8.5 (DMSO30, prepubertal donors) and 164.3±5.3 (TCM24, prepubertal donors). Inner cell mass numbers ranged between 32.0±3.0 (TCM24, adult donors) and 44.7±5.2 (cAMP30, adult donors) (S5 Table). These observations indicate apparently physiological cell numbers and allocation of cells to the two compartments of the bovine blastocyst across treatment groups. Representative pictures of blastocysts morphology prior to and after differential staining using the cAMP30 protocol is provided in S5 Fig.

### Production of progeny after transfer of embryos derived from oocytes treated with cAMP modulators

A total of 32 embryos (17 from prepubertal oocytes, 15 from adult oocytes) were non-surgically transferred (ET) to 32 recipients; after 45 days, seven (41.2%) pregnancies were determined from prepubertal females and nine pregnancies (60%) from adult donors. Ninety days after ET, six (35.3%) and seven (46.7%) pregnancies, respectively, were determined in each group. At 180 days after ET, one pregnancy was lost in the prepubertal group (29.4%). The remaining pregnancies went to term. The number of pregnancies at days 45, 90 and 180 did not differ between the type of donors (Table 2, *P* > 0.05). Five calves (4 females and one male, 53.0±2.8 kg each) were born from prepubertal oocyte donors; one died shortly after calving and cesarean section had to be performed in three cases. Seven calves were delivered from adult oocytes, including 4 males and 3 females with an average birthweight of 44.6±3.2 kg. Two of them died due to dystocia and the remaining five were delivered normally. All calves are healthy and develop normally compared with conventional artificial insemination produced calves in our animal experimental facilities.

## Discussion

The present study investigated for the first time the influence of artificially induced high cAMP levels on acquisition and maintenance of developmental capacity of oocytes collected from bovine prepubertal and adult donors using the SPOM system. Bovine oocyte and embryo preimplantation development shows striking similarities with human early development. Thus, the results may also apply to human oocyte and embryo development. Several molecular tools (gene expression and epigenetic analysis), as well as oocyte and embryo handling are well established in the bovine species, being particularly useful as a model for reproductive biology studies [23]. Moreover, prepubertal oocytes are currently used as an early source of valuable genetic material in the cattle industry.

One of the main factors involved in meiotic arrest is cyclic adenosine monophosphate (cAMP), which is produced by granulosa cells and the oocyte itself [34]. Here, intra-oocyte cAMP levels were increased dramatically in both prepubertal and adult oocytes after 2 h pre-IVM with forskolin and IBMX (prepubertal oocytes: 360.4 fmol/oocyte; adult oocytes: 396.9 fmol/oocyte) as reported previously [19]. However, after IVM intra-oocyte cAMP levels decreased, indicating that cAMP contents were not maintained at high levels during maturation in presence of cilostamide. The low cAMP levels prior to and after IVM in adult and prepubertal oocytes cultured without cAMP modulators, indicate that cAMP levels drop immediately after follicle aspiration and then remain at low levels through meiosis under *in vitro* conditions. Our results provide compelling evidence that prepubertal oocytes produce and/or receive cAMP from cumulus cells in response to cAMP modulators similar to adult oocytes, suggesting that this pathway is already functional in prepubertal bovine oocytes, which is in apparent contrast to previous reports in mice and pig prepubertal models [3, 4].

The observed increment in cAMP levels delayed progression through meiosis in oocytes obtained from prepubertal and adult donors. The maintenance of meiotic arrest was lower after 9 h in both types of oocytes than previously reported [19]. The *in vivo* oocyte retrieval method, use of heparin [35], number of cumulus cells, and inherent variation of the oocyte in the present approach may have influenced meiotic resumption and the response to cAMP modulator agents observed in this study.

An interesting side aspect of this study was that dimethyl sulfoxide (DMSO) added as a vehicle control, accelerated meiotic resumption in prepubertal and adult oocytes and reduced blastocyst yields, with a pronounced effect on prepubescent oocytes. Decreased oocyte developmental competence and lower blastocysts rates have been previously reported when DMSO was supplemented during IVM or IVC [36, 37]. Thus, DMSO even at low concentrations can affect cell integrity and normal cell function which has to be taken into account when using DMSO as solvent for defined molecules in research.

Lower *in vitro* development of oocytes derived from prepubertal donors employing 6–7 and 7–10 months old stimulated donors compared with adult donors has been previously reported [13, 14], whereas other studies using 1–4 months old unstimulated donors found similar cleavage and blastocyst rates as for adult donors [22]. Variable developmental rates have been reported using the SPOM system [27, 38], and the high success rates in the first report could not yet be confirmed, possibly due to methodological changes in culture conditions [39]. In our approach, prepubertal and adult oocytes produced similar blastocyst rates and the increased cAMP levels did not further improve *in vitro* developmental rates both in prepubertal and in adult oocytes. The prepubertal animals used in this study were ~6–9 months of age; puberty usually starts around 10–12 months in our animals. Nevertheless, we cannot rule out that at least some of the prepubertal donors were close to puberty, which may explain at least in part the similarities observed in the present study. Furthermore, it has been recently proposed in the mouse model that the follicles activated in the fetus in the ovarian medulla (first wave of primordial follicles) are actively growing after birth and remain as dominant ovulatory follicles until early adulthood [40, 41]. Oocytes isolated from these follicles could have similar or even higher developmental competence [42], which could also explain the donor similarities observed in the present study.

Satellite DNA is the most abundant fraction of the genome [43]. In the present study, CpG DNA methylation in immature oocytes was similar in all treatments for both satellite sequences. Previously, comparable results were found for BTαS, whereas BTS hypermethylation was found in prepubertal oocytes [16]. Here, increased cAMP before IVM induced hypermethylation for both satellite sequences (BTαS and BTS) in MII adult oocytes compared to *in vivo* matured oocytes, indicating that the increment in cAMP levels possibly induces methylation changes during IVM. These findings could be associated with a better synchronized de- and remethylation during embryo development, because similar CpG methylation patterns were found in blastocysts retrieved from prepubertal and adult oocytes compared to their *in vivo* produced counterparts. Genome-wide DNA demethylation occurs after fertilization and reaches the lowest level at the morula stage and remethylation is initiated in the blastocyst in a cell type specific pattern [44]. Expanded blastocysts obtained from prepubertal oocytes under standard conditions, displayed lower DNA methylation profiles than their *in vivo* counterparts for the two DNA satellites, which could suggest hypomethylation during further development. Demethylation of satellite DNA has been observed in senescent [45], cancer cells [46] and the facial anomalies syndrome (ICF) in humans [47]. Hypomethylation of satellite DNA could affect the transcriptional patterns of the entire genome via docks for transcription control proteins [48]. This hypomethylation may induce the expression of genes that are repressed under physiological conditions [49], which in turn might affect embryo development, and explain, at least in part, the differences in embryo viability reported previously for prepubertal donors [11, 12]. Hypermethylation was also observed in blastocysts produced under standard IVM conditions and in the group with DMSO supplementation suggesting gene repression and effects on further development. Our results indicate that satellite DNA methylation profiles in blastocysts can be altered by the previous oocyte culture conditions.

The relative abundance of transcripts for *PDE3A*, *ZAR1*, *PRDX1 and SLC2A8* genes was not affected by pre-IVM or *in vitro* maturation neither before nor after IVM in both types of oocytes. However, *ZAR1*, *PRDX1 and SLC2A8* transcript levels decreased markedly after maturation in all *in vitro* treatments and *in vivo* matured oocytes as previously shown for both types of donors [14, 16], indicating selective transcript degradation for these three genes during maturation and their usefulness as oocyte maturation markers. We can rule out ageing effects due to the 6 h extended IVM period as *SMAD2* levels (marker for oocyte ageing) in MII oocytes were similar among *in vitro* treatments and age groups [50, 51] and because ageing effects in bovine oocytes become apparent after 48 h of IVM [50, 52]. Furthermore, *BCL2L1*, *PRDX1*, *DNMT3b* and *SLC2A8* expression levels did not differ significantly at the blastocyst stage suggesting similar roles of these genes before and after puberty. The promoter region of *EGR1* contains two cAMP responsive elements (CRE). Protein kinase A plays an important role in activating CREB (CRE binding protein)[53]. We speculate that the observed *EGR1* up-regulation in immature prepubertal oocytes is associated with the observed increase in *PRKACA* expression, and the activation of the cAMP/PRKACA/CREB pathway by high intra-oocyte cAMP levels. Additionally, marked *EGR1* down-regulation was observed in the present study for all *in vitro* produced expanded blastocysts in contrast to adult *in vivo* produced counterparts as reported previously, suggesting a close association between *EGR1* transcription and blastocyst quality [27].

Previous studies had suggested that embryos derived from prepubertal donors lack the ability to establish and maintain a pregnancy [11, 12]. Here, we found similar rates of *in vivo* development for both age groups (prepubertal donors: 29.4%, adult donors: 46.7%), comparable to previous reports under *in vitro* standard conditions for both types of donors [26]. Obviously, the oocyte cAMP increment had no deleterious effects on the capacity of the embryos to establish and maintain pregnancy. In both donor groups some calves were found with high birthweights, which had been previously reported in calves obtained from prepubertal donors [15]. Due to the small number of transfers it is difficult to establish whether or not this observation was related to the source of oocytes, the bull used in IVF or the technique itself. Nevertheless, our results indicate that cAMP regulator agents are compatible with healthy pregnancies, established from embryos derived from adult or prepubertal donors; but care must be taken due to possible overweight of progeny.

In conclusion, we have found that bovine oocytes from prepubertal females (6–9 mo old) appear to have a functional cAMP system, similar to their adult counterparts. A detailed characterization of their cellular, molecular and epigenetic features revealed only few differences between the two types of oocytes. Cyclic AMP increment in prepubertal and adult oocytes prior to IVM delayed meiotic progression, but did not increase developmental rates. However, it shifted DNA satellite sequence methylation marks during embryo development towards that of *in vivo* derived controls. Apparently, prepubertal oocytes have a greater sensitivity to exogenous factors, such as DMSO and cAMP modulators, than their adult counterparts. Collectively, these results show that maintenance or increment of cAMP levels prior to IVM play an important role in the acquisition of full developmental competency of oocyte and embryos; but the underlying mechanisms regulating these events must be explored in future studies.

## Supporting Information

S1 Fig **Ultrasound images obtained from A) right ovary of a prepubertal bovine donor and B) right ovary from an adult bovine donor.** Every point in the scale bar on the left side indicates 0.5 cm.
(TIF)

S2 Fig **Bovine oocytes from prepubertal donors before IVM: A) cAMP30 protocol, B) DMSO30 protocol, C) TCM24 protocol and after IVM: D) cAMP30 protocol, E) DMSO30 protocol, and F) TCM24 protocol.** Different categories of oocytes before IVM are shown. Scale bar = 500 μm.
(TIF)

S3 Fig **Bovine oocytes from adult donors before IVM: A) cAMP30 protocol, B) DMSO30 protocol, C) TCM24 protocol and after IVM: D) cAMP30 protocol, E) DMSO30 protocol, and F) TCM24 protocol.** Different categories of oocytes before IVM are shown. Scale bar = 500 μm.
(TIF)

S4 Fig Progression of the meiosis in bovine oocytes from prepubertal and adult donors using the cAMP30 protocol. Germinal vesicle (A, B), germinal vesicle breakdown (C, D), metaphase I (E, F) and metaphase II (G, H) status. Scale bar = 500 μm. Scale bar = 100 μm.
(TIF)

S5 Fig Day 8 bovine expanded blastocysts obtained from prepubertal and *a*dult oocytes matured using the cAMP30 protocol. Blastocysts before (A, B) and after differential staining (C, D). The trophectoderm nuclei are shown in red and the inner cell mass nuclei in blue. Scale bar = 100 μm.
(TIF)

S1 Table Primer sequences used in RT-qPCR for selected genes. (DOCX)

S2 Table Primers used for amplification and sequencing of satellite sequences in immature oocytes, MII oocytes and expanded blastocysts. (DOCX)

S3 Table Progression through meiosis of oocytes retrieved from adult and prepubescent donors and treated pre and during IVM with and without cAMP modulators. (DOCX)

S4 Table Number of CpG and methylation profiles of two satellite sequences in immature oocytes, MII oocytes and expanded blastocysts obtained after prepubertal and adult oocyte treatment with cAMP modulators. (DOCX)

S5 Table Blastocyst cell numbers in expanded blastocysts derived from adult and prepubescent oocytes treated pre and during IVM with cAMP modulators. (DOCX)

## References

[1] Shuhaibar LC, Egbert JR, Norris RP, Lampe PD, Nikolaev VO, Thunemann M, et al Intercellular signaling via cyclic GMP diffusion through gap junctions restarts meiosis in mouse ovarian follicles. Proc Natl Acad Sci U S A. 2015;112(17):5527–32. Epub 2015/03/17. 10.1073/pnas.1423598112 ; PubMed Central PMCID: PMCPmc4418852. 25775542 PMC4418852

[2] Wang Y, Teng Z, Li G, Mu X, Wang Z, Feng L, et al Cyclic AMP in oocytes controls meiotic prophase I and primordial folliculogenesis in the perinatal mouse ovary. Development. 2015;142(2):343–51. Epub 2014/12/17. 10.1242/dev.112755 . 25503411

[3] Bagg MA, Nottle MB, Grupen CG, Armstrong DT. Effect of dibutyryl cAMP on the cAMP content, meiotic progression, and developmental potential of *in vitro* matured pre-pubertal and adult pig oocytes. Mol Reprod Dev. 2006;73(10):1326–32. Epub 2006/07/26. 10.1002/mrd.20555 . 16865720

[4] Han D, Cao XY, Wang HL, Li JJ, Wang YB, Tan JH. Effects of puberty and gonadotropins on the molecular events controlling meiotic resumption of mouse oocytes. Reproduction. 2010;139(6):959–69. Epub 2010/04/14. 10.1530/rep-09-0485 . 20385781

[5] Bruot BC, Gearing M, Musey PI, Wilson ME, Collins DC. Steroidogenesis by the immature rhesus monkey ovary *in vitro*. J Endocrinol Invest. 1986;9(2):171–5. Epub 1986/04/01. 10.1007/bf03348091 . 3011886

[6] Ledda S, Bogliolo L, Leoni G, Naitana S. Cell coupling and maturation-promoting factor activity in *in vitro*-matured prepubertal and adult sheep oocytes. Biol Reprod. 2001;65(1):247–52. Epub 2001/06/23. . 11420246 10.1095/biolreprod65.1.247

[7] Tsafriri A, Pomerantz SH. Oocyte maturation inhibitor. Clin Endocrinol Metab. 1986;15(1):157–70. Epub 1986/02/01. . 3514001 10.1016/s0300-595x(86)80047-0

[8] Pincus G, Enzmann EV. The Comparative Behavior of Mammalian Eggs *in vivo* and *in vitro*: I. The Activation of Ovarian Eggs. J Exp Med. 1935;62(5):665–75. Epub 1935/10/31. 19870440 10.1084/jem.62.5.665 PMC2133299

[9] Rizos D, Ward F, Duffy P, Boland MP, Lonergan P. Consequences of bovine oocyte maturation, fertilization or early embryo development *in vitro* versus *in vivo*: implications for blastocyst yield and blastocyst quality. Mol Reprod Dev. 2002;61(2):234–48. Epub 2002/01/23. 10.1002/mrd.1153 . 11803560

[10] Armstrong DT, Irvine BJ, Earl CR, McLean D, Seamark RF. Gonadotropin stimulation regimens for follicular aspiration and *in vitro* embryo production from calf oocytes. Theriogenology. 1994;42(7):1227–36. Epub 1994/01/01. . 16727627 10.1016/0093-691x(94)90871-0

[11] Fry RC, Simpson TL, Squires TJ. Ultrasonically guided transvaginal oocyte recovery from calves treated with or without GnRH. Theriogenology. 1998;49(6):1077–82. . 10732047 10.1016/s0093-691x(98)00057-0

[12] Revel F, Mermillod P, Peynot N, Renard JP, Heyman Y. Low developmental capacity of *in vitro* matured and fertilized oocytes from calves compared with that of cows. J Reprod Fertil. 1995;103(1):115–20. Epub 1995/01/01. . 7707286 10.1530/jrf.0.1030115

[13] Oropeza A, Wrenzycki C, Herrmann D, Hadeler KG, Niemann H. Improvement of the developmental capacity of oocytes from prepubertal cattle by intraovarian insulin-like growth factor-I application. Biol Reprod. 2004;70(6):1634–43. Epub 2004/02/10. 10.1095/biolreprod.103.025494 . 14766727

[14] Zaraza J, Oropeza A, Velazquez MA, Korsawe K, Herrmann D, Carnwath JW, et al Developmental competence and mRNA expression of preimplantation *in vitro*-produced embryos from prepubertal and postpubertal cattle and their relationship with apoptosis after intraovarian administration of IGF-1. Theriogenology. 2010;74(1):75–89. Epub 2010/02/09. 10.1016/j.theriogenology.2009.11.033 . 20138354

[15] Taneja M, Bols PEJ, de Velde AV, Ju J-C, Schreiber D, Tripp MW, et al Developmental Competence of Juvenile Calf Oocytes *In vitro* and *In vivo*: Influence of Donor Animal Variation and Repeated Gonadotropin Stimulation. Biol Reprod. 2000;62(1):206–13. 10.1095/biolreprod62.1.206 10611087

[16] Diederich M, Hansmann T, Heinzmann J, Barg-Kues B, Herrmann D, Aldag P, et al DNA methylation and mRNA expression profiles in bovine oocytes derived from prepubertal and adult donors. Reproduction. 2012;144(3):319–30. Epub 2012/06/27. 10.1530/REP-12-0134 . 22733804

[17] Grupen CG, Fung M, Armstrong DT. Effects of milrinone and butyrolactone-I on porcine oocyte meiotic progression and developmental competence. Reprod Fertil Dev. 2006;18(3):309–17. Epub 2006/03/24. . 16554006 10.1071/rd05125

[18] Albarracin JL, Morato R, Izquierdo D, Mogas T. Effects of roscovitine on the nuclear and cytoskeletal components of calf oocytes and their subsequent development. Theriogenology. 2005;64(8):1740–55. Epub 2005/06/07. 10.1016/j.theriogenology.2005.04.018 . 15936813

[19] Albuz FK, Sasseville M, Lane M, Armstrong DT, Thompson JG, Gilchrist RB. Simulated physiological oocyte maturation (SPOM): a novel *in vitro* maturation system that substantially improves embryo yield and pregnancy outcomes. Hum Reprod. 2010;25(12):2999–3011. Epub 2010/09/28. 10.1093/humrep/deq246 . 20870682

[20] Oktay K, Bedoschi G, Berkowitz K, Bronson R, Kashani B, McGovern P, et al Fertility Preservation in Females with Turner Syndrome: A Comprehensive Review and Practical Guidelines. J Pediatr Adolesc Gynecol. doi: 10.1016/j.jpag.2015.10.011. PMC5015771 26485320

[21] Estes SJ. Fertility Preservation in Children and Adolescents. Endocrinol Metab Clin North Am. 2015;44(4):799–820. Epub 2015/11/17. 10.1016/j.ecl.2015.07.005 . 26568494

[22] Kauffold J, Amer HA, Bergfeld U, Weber W, Sobiraj A. The *in vitro* developmental competence of oocytes from juvenile calves is related to follicular diameter. J Reprod Dev. 2005;51(3):325–32. Epub 2005/07/08. . 16000866 10.1262/jrd.17002

[23] Urrego R, Rodriguez-Osorio N, Niemann H. Epigenetic disorders and altered gene expression after use of Assisted Reproductive Technologies in domestic cattle. Epigenetics. 2014;9(6):803–15. Epub 2014/04/09. 10.4161/epi.28711 24709985 PMC4065177

[24] Parrish JJ, Susko-Parrish J, Winer MA, First NL. Capacitation of bovine sperm by heparin. Biol Reprod. 1988;38(5):1171–80. 10.1095/biolreprod38.5.1171 3408784

[25] Bungartz L, Niemann H. Assessment of the presence of a dominant follicle and selection of dairy cows suitable for superovulation by a single ultrasound examination. J Reprod Fertil. 1994;101(3):583–91. Epub 1994/08/01. . 7966012 10.1530/jrf.0.1010583

[26] Khatir H, Lonergan P, Touze JL, Mermillod P. The characterization of bovine embryos obtained from prepubertal calf oocytes and their viability after non surgical embryo transfer. Theriogenology. 1998;50(8):1201–10. Epub 2000/03/29. . 10734435 10.1016/s0093-691x(98)00220-9

[27] Bernal SM, Heinzmann J, Herrmann D, Timmermann B, Baulain U, Großfeld R, et al Effects of different oocyte retrieval and *in vitro* maturation systems on bovine embryo development and quality. Zygote. 2014;FirstView:1–11. 10.1017/S0967199413000658 24423448

[28] Niemann H, Carnwath JW, Herrmann D, Wieczorek G, Lemme E, Lucas-Hahn A, et al DNA methylation patterns reflect epigenetic reprogramming in bovine embryos. Cell Reprogram. 2010;12(1):33–42. 10.1089/cell.2009.0063 . 20132011

[29] Kang YK, Lee HJ, Shim JJ, Yeo S, Kim SH, Koo DB, et al Varied patterns of DNA methylation change between different satellite regions in bovine preimplantation development. Mol Reprod Dev. 2005;71(1):29–35. Epub 2005/03/01. 10.1002/mrd.20249 . 15736134

[30] Bock C, Reither S, Mikeska T, Paulsen M, Walter J, Lengauer T. BiQ Analyzer: visualization and quality control for DNA methylation data from bisulfite sequencing. Bioinformatics. 2005;21(21):4067–8. Epub 2005/09/06. 10.1093/bioinformatics/bti652 . 16141249

[31] Thouas GA, Korfiatis NA, French AJ, Jones GM, Trounson AO. Simplified technique for differential staining of inner cell mass and trophectoderm cells of mouse and bovine blastocysts. Reprod Biomed Online. 2001;3(1):25–9. Epub 2003/01/07. . 12513888 10.1016/s1472-6483(10)61960-8

[32] R Development Core Team. R: A language and environment for statistical computing Vienna, Austria: R Foundation for Statistical Computing; 2014.

[33] SAS Institute. The SAS system for Windows. Release 9.4. Cary, NC, USA: SAS Int.; 2008.

[34] Coticchio G, Dal Canto M, Mignini Renzini M, Guglielmo MC, Brambillasca F, Turchi D, et al Oocyte maturation: gamete-somatic cells interactions, meiotic resumption, cytoskeletal dynamics and cytoplasmic reorganization. Hum Reprod Update. 2015 10.1093/humupd/dmv011 . 25744083

[35] Zeng HT, Ren Z, Guzman L, Wang X, Sutton-McDowall ML, Ritter LJ, et al Heparin and cAMP modulators interact during pre-*in vitro* maturation to affect mouse and human oocyte meiosis and developmental competence. Hum Reprod. 2013;28(6):1536–45. Epub 2013/04/06. 10.1093/humrep/det086 . 23559189

[36] Avery B, Greve T. Effects of ethanol and dimethylsulphoxide on nuclear and cytoplasmic maturation of bovine cumulus-oocyte complexes. Mol Reprod Dev. 2000;55(4):438–45. Epub 2000/03/01. 10.1002/(sici)1098-2795(200004)55:4<438::aid-mrd12>3.0.co;2-y . 10694752

[37] Stinshoff H, Wilkening S, Hanstedt A, Bollwein H, Wrenzycki C. Dimethylsulfoxide and conjugated linoleic acids affect bovine embryo development *in vitro*. Reprod Fertil Dev. 2014;26(4):502–10. Epub 2013/04/12. 10.1071/rd12372 . 23574633

[38] Guimaraes AL, Pereira SA, Leme LO, Dode MA. Evaluation of the simulated physiological oocyte maturation system for improving bovine *in vitro* embryo production. Theriogenology. 2015;83(1):52–7. Epub 2014/12/03. 10.1016/j.theriogenology.2014.07.042 . 25447152

[39] Gilchrist RB, Zeng HT, Wang X, Richani D, Smitz J, Thompson JG. Reevaluation and evolution of the simulated physiological oocyte maturation system. Theriogenology. 2015;84(4):656–7. Epub 2015/05/11. 10.1016/j.theriogenology.2015.03.032 . 25958085

[40] Zheng W, Zhang H, Gorre N, Risal S, Shen Y, Liu K. Two classes of ovarian primordial follicles exhibit distinct developmental dynamics and physiological functions. Hum Mol Genet. 2014;23(4):920–8. Epub 2013/10/03. 10.1093/hmg/ddt486 ; PubMed Central PMCID: PMCPmc3900105. 24087793 PMC3900105

[41] Mork L, Maatouk DM, McMahon JA, Guo JJ, Zhang P, McMahon AP, et al Temporal differences in granulosa cell specification in the ovary reflect distinct follicle fates in mice. Biol Reprod. 2012;86(2):37 Epub 2011/10/07. 10.1095/biolreprod.111.095208 ; PubMed Central PMCID: PMCPmc3290667. 21976597 PMC3290667

[42] Zheng W, Zhang H, Liu K. The two classes of primordial follicles in the mouse ovary: their development, physiological functions and implications for future research. Mol Hum Reprod. 2014;20(4):286–92. Epub 2014/01/23. 10.1093/molehr/gau007 . 24448914

[43] Enukashvily NI, Ponomartsev NV. Mammalian satellite DNA: a speaking dumb. Adv Protein Chem Struct Biol. 2013;90:31–65. Epub 2013/04/16. 10.1016/b978-0-12-410523-2.00002-x . 23582201

[44] Wu H, Zhang Y. Reversing DNA methylation: mechanisms, genomics, and biological functions. Cell. 2014;156(1–2):45–68. Epub 2014/01/21. 10.1016/j.cell.2013.12.019 24439369 PMC3938284

[45] Suzuki T, Fujii M, Ayusawa D. Demethylation of classical satellite 2 and 3 DNA with chromosomal instability in senescent human fibroblasts. Exp Gerontol. 2002;37(8–9):1005–14. Epub 2002/09/06. . 12213551 10.1016/s0531-5565(02)00061-x

[46] Wilson AS, Power BE, Molloy PL. DNA hypomethylation and human diseases. Biochim Biophys Acta. 2007;1775(1):138–62. Epub 2006/10/19. 10.1016/j.bbcan.2006.08.007 . 17045745

[47] Brun ME, Lana E, Rivals I, Lefranc G, Sarda P, Claustres M, et al Heterochromatic genes undergo epigenetic changes and escape silencing in immunodeficiency, centromeric instability, facial anomalies (ICF) syndrome. PLoS One. 2011;6(4):e19464 Epub 2011/05/12. 10.1371/journal.pone.0019464 ; PubMed Central PMCID: PMCPmc3084872. 21559330 PMC3084872

[48] Ehrlich M. DNA hypomethylation in cancer cells. Epigenomics. 2009;1(2):239–59. Epub 2010/05/25. 10.2217/epi.09.33 ; PubMed Central PMCID: PMCPmc2873040. 20495664 PMC2873040

[49] Gopalakrishnan S, Sullivan BA, Trazzi S, Della Valle G, Robertson KD. DNMT3B interacts with constitutive centromere protein CENP-C to modulate DNA methylation and the histone code at centromeric regions. Hum Mol Genet. 2009;18(17):3178–93. Epub 2009/06/02. 10.1093/hmg/ddp256 ; PubMed Central PMCID: PMCPmc2722982. 19482874 PMC2722982

[50] Zhang G-M, Gu C-H, Zhang Y-L, Sun H-Y, Qian W-P, Zhou Z-R, et al Age-associated changes in gene expression of goat oocytes. Theriogenology. 2013;80(4):328–36. 10.1016/j.theriogenology.2013.04.019 23746875

[51] Grondahl ML, Yding Andersen C, Bogstad J, Nielsen FC, Meinertz H, Borup R. Gene expression profiles of single human mature oocytes in relation to age. Hum Reprod. 2010;25(4):957–68. Epub 2010/02/12. 10.1093/humrep/deq014 . 20147335

[52] Heinzmann J, Mattern F, Aldag P, Bernal-Ulloa SM, Schneider T, Haaf T, et al Extended *in vitro* maturation affects gene expression and DNA methylation in bovine oocytes. Mol Hum Reprod. 2015;21(10):770–82. Epub 2015/07/15. 10.1093/molehr/gav040 . 26155800

[53] Li B, Kaetzel MA, Dedman JR. Signaling pathways regulating murine cardiac CREB phosphorylation. Biochem Biophys Res Commun. 2006;350(1):179–84. Epub 2006/09/26. 10.1016/j.bbrc.2006.09.016 . 16996475

