# Supplementary figures and images for: Cyclic AMP Affects Oocyte Maturation and Embryo Development in Prepubertal and Adult Cattle
Source: PLoS One. 2016 Feb 29;11(2):e0150264. doi: 10.1371/journal.pone.0150264 (PMC4771806; doi:10.1371/journal.pone.0150264)

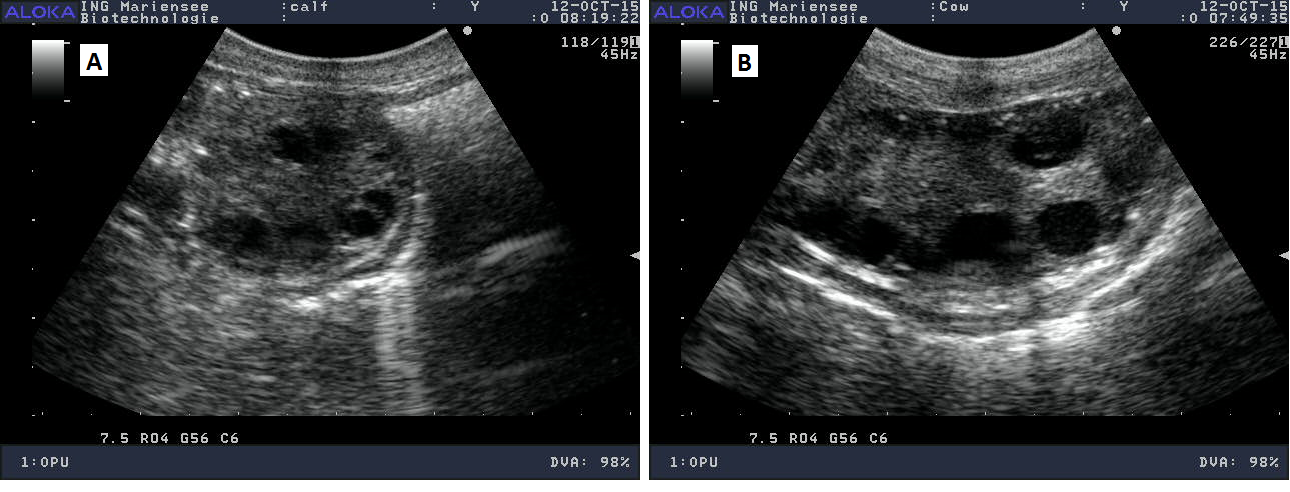

Supplement: S1 Fig — Ultrasound images obtained from A) right ovary of a prepubertal bovine donor and B) right ovary from an adult bovine donor. Every point in the scale bar on the left side indicates 0.5 cm. (TIF) [file pone.0150264.s001.tif]

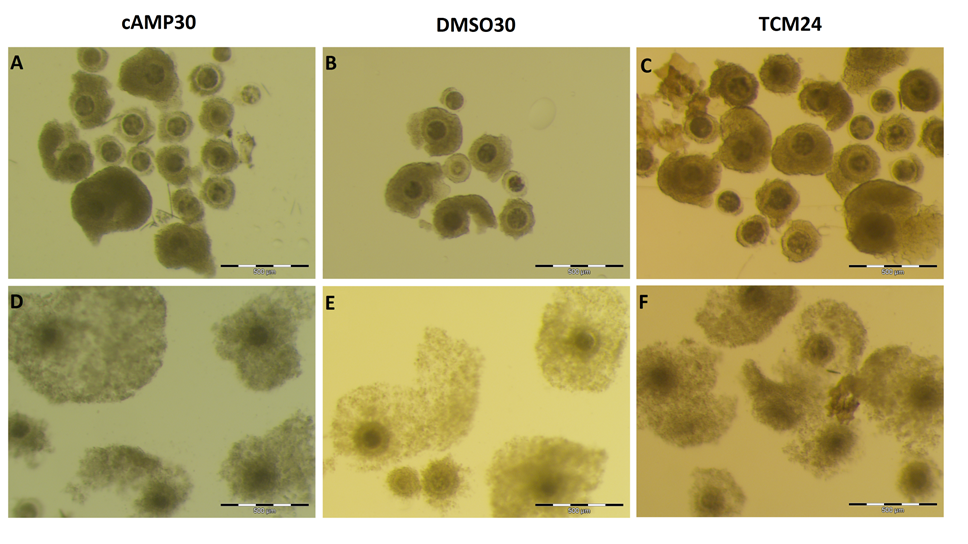

Supplement: S2 Fig — Bovine oocytes from prepubertal donors before IVM: A) cAMP30 protocol, B) DMSO30 protocol, C) TCM24 protocol and after IVM: D) cAMP30 protocol, E) DMSO30 protocol, and F) TCM24 protocol. Different categories of oocytes before IVM are shown. Scale bar = 500 μm. (TIF) [file pone.0150264.s002.tif]

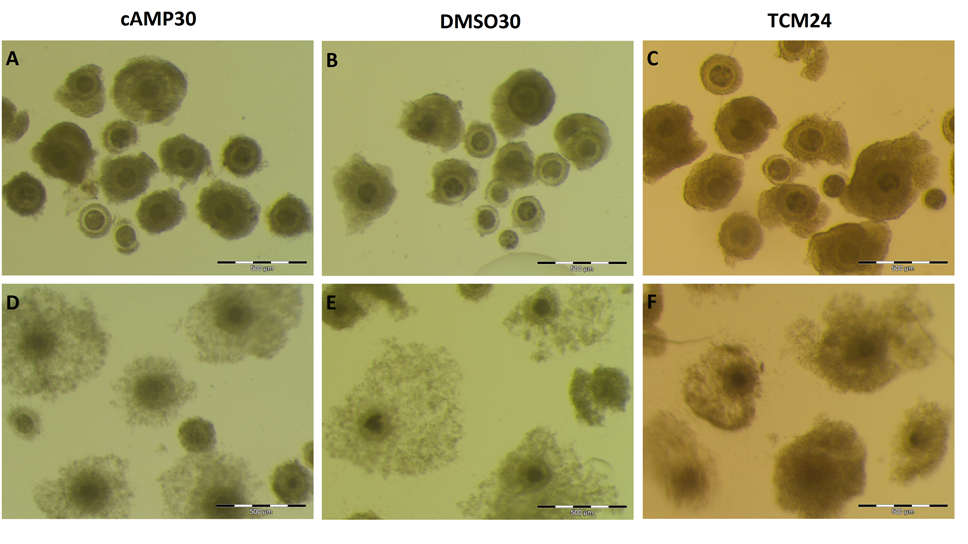

Supplement: S3 Fig — Bovine oocytes from adult donors before IVM: A) cAMP30 protocol, B) DMSO30 protocol, C) TCM24 protocol and after IVM: D) cAMP30 protocol, E) DMSO30 protocol, and F) TCM24 protocol. Different categories of oocytes before IVM are shown. Scale bar = 500 μm. (TIF) [file pone.0150264.s003.tif]

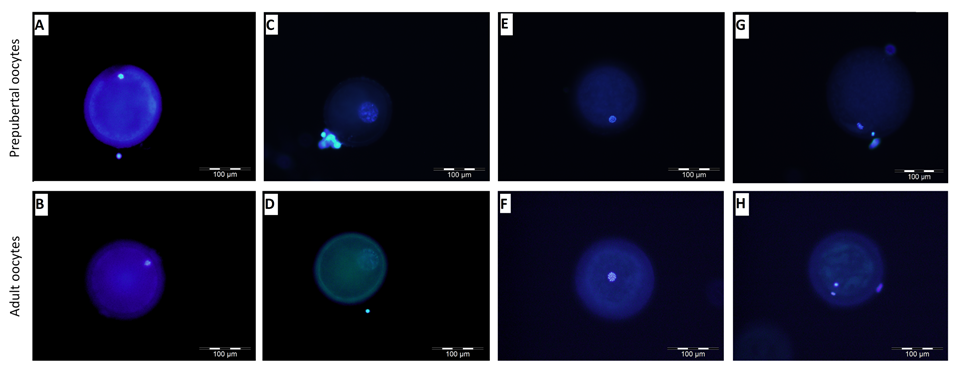

Supplement: S4 Fig — Germinal vesicle (A, B), germinal vesicle breakdown (C, D), metaphase I (E, F) and metaphase II (G, H) status. Scale bar = 500 μm. Scale bar = 100 μm. (TIF) [file pone.0150264.s004.tif]

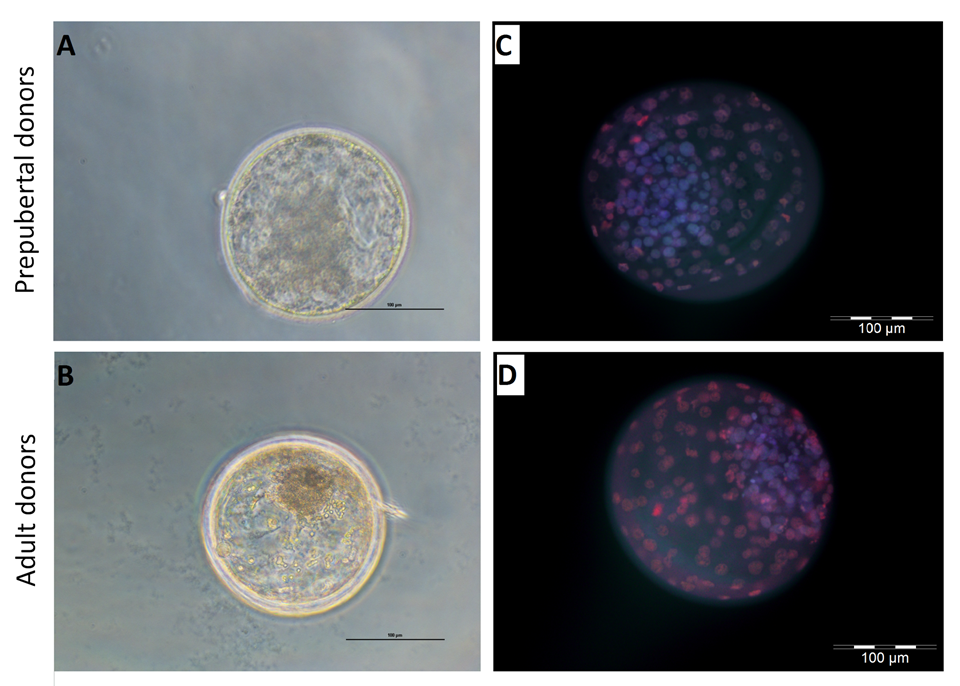

Supplement: S5 Fig — Blastocysts before (A, B) and after differential staining (C, D). The trophectoderm nuclei are shown in red and the inner cell mass nuclei in blue. Scale bar = 100 μm. (TIF) [file pone.0150264.s005.tif]
