# Supplementary material for: Cyclic AMP Affects Oocyte Maturation and Embryo Development in Prepubertal and Adult Cattle
Source: PLoS One. 2016 Feb 29;11(2):e0150264. doi: 10.1371/journal.pone.0150264 (PMC4771806; doi:10.1371/journal.pone.0150264)
Supplement: S1 Table — (DOCX) [file pone.0150264.s006.docx]

S1 Table. Primer sequences used in RT-qPCR for selected genes.

| Gene identification | Primer sequences (5´-3´, forward and reverse) | Location | GenBank accession number | Product length (bp) | Reference |
| --- | --- | --- | --- | --- | --- |
| BCL2-like 1 (BCL2L1) | F: GGTGAGTCGGATCGCAACTT | BCL2L1_fw (486–505) | AB238936.1 | 70 | [1] |
|  | R: GTTCTCCTGGATCCAAGGCTCTA | BCL2L1_rev (533–555) |  |  |  |
| DNA cytosine-5 methyltransferase3b (DNMT3b) | F: GGCCTCGGAAGTGTGTGA | DNMT3B_fw (1779–1796) | NM_181813.2 | 62 | [2] |
|  | R: TGATGTTGCCCTCGTGCTTA | DNMT3B_rev (1821–1840) |  |  |  |
| Early growth response 1 (EGR1) | F: GTGCAATTGTGAGGGATGTG | EGR1_fw (2695-2714) | BC118328 | 88 | [3] |
|  | R: TCCTTGTTTTGGCTCCCAAAG | EGR1_rev (2763-2782) |  |  |  |
| Globin (Rabbit alpha-1 globin) | F: GCAGCCACGGTGGCGAGTAT | Globin_fw (92-111) | NM_001082389.2 | 257 | [4] |
|  | R: GTGGGACAGGAGCTTGAAAT | Globin_rev (329-348) |  |  |  |
| Phosphodiesterase 3A (PDE3A) | F: TTCAGTGGACGGAGGGTATTGT | PDE3A_fw (116-137) | XM_002687752.2 | 98 | This work |
|  | R: AGCGGAACGGTCCATGAAG | PDE3A_rev (195-213) |  |  |  |
| Peroxiredoxin 1 (PRDX1) | F: TCAAGCCTGATGTCCAGAAGAGC | PRDX1_fw (593-615) | NM_174431.1 | 174 | [5] |
|  | R: CCGTCCTGTCCCACACCAC | PRDX1_rev (748-766) |  |  |  |
| Protein kinase cAMP-activated catalytic subunit alpha (PRKACA) | F: TCTGGGAAGGTGCGGTTTC | PRKACA_fw (115-133) | NM_174584.2 | 83 | This work |
|  | R: TTGGTGAGGTCCACTTGTAGGA | PRKACA_rev (176-197) |  |  |  |
| SMAD family member 2 (SMAD2) | F: ACCCTTACCACTACCAGAGAGTTGA | SMAD2_fw (162-186) | NM_001046218 | 83 | This work |
|  | R: CAGTTCCGTTAGGATCTCGGTATG | SMAD2_rev (221-244) |  |  |  |
| SLC2A8 (GLUT8) (Solute carrier family 2 facilitated glucose transporter, member 8) | F: GCATCTTCGGTGTCCTTTTCA | GLUT8_fw (1441–1461) | AY208940 | 81 | [2] |
|  | R: CAAAATGGGCTGTGATTTGCT | GLUT8_rev (1501–1521) |  |  |  |
| Zygote arrest 1 (ZAR1) | F: AGACTAGATGCTCCTGCCCAGT | ZAR1_fw (1022-1043) | NM_001076203.1 | 64 | [6] |
|  | R: TCTTGAGGGTGGGGCCGTTTAG | ZAR1_rev (1064-1085) |  |  |  |

1. Zaraza J, Oropeza A, Velazquez MA, Korsawe K, Herrmann D, Carnwath JW, et al. Developmental competence and mRNA expression of preimplantation in vitro-produced embryos from prepubertal and postpubertal cattle and their relationship with apoptosis after intraovarian administration of IGF-1. Theriogenology. 2010;74(1):75-89. Epub 2010/02/09. doi: 10.1016/j.theriogenology.2009.11.033. PubMed PMID: 20138354.

2. Heinzmann J, Hansmann T, Herrmann D, Wrenzycki C, Zechner U, Haaf T, et al. Epigenetic profile of developmentally important genes in bovine oocytes. Mol Reprod Dev. 2011;78(3):188-201. Epub 2011/02/04. doi: 10.1002/mrd.21281. PubMed PMID: 21290475.

3. Bernal SM, Heinzmann J, Herrmann D, Timmermann B, Baulain U, Großfeld R, et al. Effects of different oocyte retrieval and in vitro maturation systems on bovine embryo development and quality. Zygote. 2014;FirstView:1-11. doi: doi:10.1017/S0967199413000658.

4. Cheng JF, Raid L, Hardison RC. Block duplications of a zeta-zeta-alpha-theta gene set in the rabbit alpha-like globin gene cluster. J Biol Chem. 1987;262(11):5414-21. Epub 1987/04/15. PubMed PMID: 3031053.

5. Thelie A, Papillier P, Pennetier S, Perreau C, Traverso J, Uzbekova S, et al. Differential regulation of abundance and deadenylation of maternal transcripts during bovine oocyte maturation in vitro and in vivo. BMC Dev Biol. 2007;7(1):125. PubMed PMID: doi:10.1186/1471-213X-7-125.

6. Diederich M, Hansmann T, Heinzmann J, Barg-Kues B, Herrmann D, Aldag P, et al. DNA methylation and mRNA expression profiles in bovine oocytes derived from prepubertal and adult donors. Reproduction. 2012;144(3):319-30. Epub 2012/06/27. doi: 10.1530/REP-12-0134. PubMed PMID: 22733804.
