# Supplementary material for: Cyclic AMP Affects Oocyte Maturation and Embryo Development in Prepubertal and Adult Cattle
Source: PLoS One. 2016 Feb 29;11(2):e0150264. doi: 10.1371/journal.pone.0150264 (PMC4771806; doi:10.1371/journal.pone.0150264)
Supplement: S2 Table — (DOCX) [file pone.0150264.s007.docx]

S2 Table. Primers used for amplification and sequencing of satellite sequences in immature oocytes, MII oocytes and expanded blastocysts.

| Repeat/  binding site | GenBank accession number | Primer sequences (5´- 3´) | Fragment size | Reference |
| --- | --- | --- | --- | --- |
| Bovine testis satellite I (BTS) | J00032.1 | F: AATACCTCTAATTTCAAACT | 211bp | [1] |
|  |  | R: TTTGTGAATGTAGTTAATA |  |  |
| Bovine Taurus alpha satellite I (BTαS) | AJ293510.1 | F: GATGTTTTYGGGGAGAGAGG | 154bp | [1] |
|  |  | R: CCRATCCCCTCTTAATAAAAACC |  |  |
| T7 |  | ACTCACTATAGGGCGAATTG |  | [2] |
| SP6 |  | ATTTAGGTGACACTATAGAATACTC |  | [2] |

1. Kang YK, Lee HJ, Shim JJ, Yeo S, Kim SH, Koo DB, et al. Varied patterns of DNA methylation change between different satellite regions in bovine preimplantation development. Mol Reprod Dev. 2005;71(1):29-35. Epub 2005/03/01. doi: 10.1002/mrd.20249. PubMed PMID: 15736134.

2. Diederich M, Hansmann T, Heinzmann J, Barg-Kues B, Herrmann D, Aldag P, et al. DNA methylation and mRNA expression profiles in bovine oocytes derived from prepubertal and adult donors. Reproduction. 2012;144(3):319-30. Epub 2012/06/27. doi: 10.1530/REP-12-0134. PubMed PMID: 22733804.
