# Supplementary material for: Cyclic AMP Affects Oocyte Maturation and Embryo Development in Prepubertal and Adult Cattle
Source: PLoS One. 2016 Feb 29;11(2):e0150264. doi: 10.1371/journal.pone.0150264 (PMC4771806; doi:10.1371/journal.pone.0150264)
Supplement: S3 Table — (DOCX) [file pone.0150264.s008.docx]

**S3 Table. Progression through meiosis of oocytes retrieved from adult and prepubescent donors and treated pre and during IVM with and without cAMP modulators.**

| Donors | Treatment | Time after IVM (h) | Oocyte stage | | | | | | | | | | Total  (n) |
| --- | --- | --- | --- | --- | --- | --- | --- | --- | --- | --- | --- | --- | --- |
|  |  |  | GV (n) | GV (%) | GVBD (n) | GVBD (%) | MI (n) | MI (%) | MII (n) | MII (%) | Not evaluable (n) | Not  evaluable (%) |  |
| Prepubertal | cAMP30 | 9 | 47 | 68.1^x^ | 20 | 29.0 | 1 | 1.4^x^ | 0 | 0.0 | 1 | 1.4 | 69 |
| Prepubertal | DMSO30 | 9 | 15 | 21.7^y^ | 23 | 33.3 | 30 | 43.5^y^ | 1 | 1.4 | 0 | 0.0 | 69 |
| Prepubertal | TCM24 | 9 | 29 | 46.0^z^ | 17 | 27.0 | 17 | 27.0^z^ | 0 | 0.0 | 0 | 0.0 | 63 |
| Adult | cAMP30 | 9 | 35 | 55.6^a^ | 28 | 44.4^a^ | 0 | 0.0^a^ | 0 | 0.0 | 0 | 0.0 | 63 |
| Adult | DMSO30 | 9 | 21 | 31.3^b^ | 25 | 37.3^b^ | 17 | 25.4^b^ | 2 | 3.0 | 2 | 3.0 | 67 |
| Adult | TCM24 | 9 | 25 | 46.3^c^ | 20 | 37.0^b^ | 9 | 16.7^b^ | 0 | 0.0 | 0 | 0.0 | 54 |
| Prepubertal | cAMP30 | 20 | 7 | 9.9^x^ | 16 | 22.5^x^ | 36 | 50.7^x^ | 11 | 15.5^x^ | 1 | 1.4 | 71 |
| Prepubertal | DMSO30 | 20 | 2 | 2.7^y^ | 3 | 4.1^y^ | 22 | 30.1^y^ | 44 | 60.3^y^ | 2 | 2.7 | 73 |
| Prepubertal | TCM24 | 20 | 2 | 2.9^z^ | 5 | 7.2^y^ | 20 | 29.0^y^ | 41 | 59.4^y^ | 1 | 1.4 | 69 |
| Adult | cAMP30 | 20 | 7 | 10.6^a^ | 15 | 22.7^a^ | 36 | 54.5^a^ | 6 | 9.1^a^ | 2 | 3.0 | 66 |
| Adult | DMSO30 | 20 | 1 | 1.4^b^ | 0 | 0.0^b^ | 17 | 24.6^b^ | 50 | 72.5^b^ | 1 | 1.4 | 69 |
| Adult | TCM24 | 20 | 0 | 0.0^b^ | 2 | 3.4^b^ | 18 | 30.5^b^ | 37 | 62.7^b^ | 2 | 3.4 | 59 |
| Prepubertal | cAMP30 | 30 | 0 | 0.0 | 7 | 10.8^x^ | 15 | 23.1^x^ | 41 | 63.1^x^ | 2 | 3.1 | 65 |
| Prepubertal | DMSO30 | 30 | 2 | 2.6 | 0 | 0.0^y^ | 9 | 11.5^y^ | 65 | 83.3^y^ | 2 | 2.6 | 78 |
| Prepubertal | TCM24 | 24 | 0 | 0.0 | 1 | 1.7^y^ | 5 | 8.5^y^ | 52 | 88.1^y^ | 1 | 1.7 | 59 |
| Adult | cAMP30 | 30 | 0 | 0.0 | 4 | 6.5 | 1 | 1.6^a^ | 57 | 91.9^a^ | 0 | 0.0 | 62 |
| Adult | DMSO30 | 30 | 0 | 0.0 | 2 | 2.7 | 5 | 6.8^ab^ | 66 | 89.2^a^ | 1 | 1.4 | 74 |
| Adult | TCM24 | 24 | 0 | 0.0 | 2 | 3.6 | 7 | 12.5^b^ | 44 | 78.6^b^ | 3 | 5.4 | 56 |

Data are the total number of oocytes per treatment per fixation time from four replicates; *P* < 0.016. GV, germinal vesicle stage; GVBD, germinal vesicle breakdown; MI, metaphase I; MII, metaphase II. Values with different superscripts differ significantly for the respective IVM time for prepubertal (x, y, z) and adult donors (a, b, c).
