# Supplementary material for: Cyclic AMP Affects Oocyte Maturation and Embryo Development in Prepubertal and Adult Cattle
Source: PLoS One. 2016 Feb 29;11(2):e0150264. doi: 10.1371/journal.pone.0150264 (PMC4771806; doi:10.1371/journal.pone.0150264)
Supplement: S4 Table — (DOCX) [file pone.0150264.s009.docx]

**S4 Table. Number of CpG and methylation profiles of two satellite sequences in immature oocytes, MII oocytes and expanded blastocysts obtained after prepubertal and adult oocyte treatment with cAMP modulators.**

|  |  |  |  | Type of sample | | | | | | | | | | | | | |
| --- | --- | --- | --- | --- | --- | --- | --- | --- | --- | --- | --- | --- | --- | --- | --- | --- | --- |
|  |  |  |  | Immature oocytes | | | |  | MII oocytes | | | |  | Expanded blastocyst | | | |
| Satellite | Donors | Treatment |  | Sequenced clones (n) | CpGs evaluated (n) | CpGs methylated (n) | Methylation level (%) |  | Sequenced clones (n) | CpGs evaluated (n) | CpGs methylated (n) | Methylation level (%) |  | Sequenced clones (n) | CpGs evaluated (n) | CpGs methylated (n) | Methylation level (%) |
| Bovine testis satellite I (BTS) | Prepubertal | cAMP30 |  | 29 | 331 | 209 | 63.1 |  | 29 | 342 | 207 | 60.5 ^ab^ |  | 47 | 550 | 71 | 12.9 ^ab^ |
|  |  | DMSO30 |  | 28 | 326 | 180 | 55.2 |  | 29 | 340 | 192 | 56.5 ^b^ |  | 46 | 542 | 96 | 17.7 ^a^ |
|  |  | TCM24 |  | 29 | 331 | 198 | 59.8 |  | 30 | 348 | 203 | 58.3 ^ab^ |  | 44 | 515 | 17 | 3.3 ^c^ |
|  | Adult | cAMP30 |  | 29 | 340 | 210 | 61.8 |  | 29 | 338 | 230 | 68.0 ^a^ |  | 48 | 562 | 82 | 14.6 ^ab^ |
|  |  | DMSO30 |  | 39 | 454 | 283 | 62.3 |  | 31 | 365 | 217 | 59.5 ^ab^ |  | 47 | 550 | 53 | 9.6 ^b^ |
|  |  | TCM24 |  | 29 | 336 | 216 | 64.3 |  | 30 | 353 | 209 | 59.2 ^ab^ |  | 49 | 574 | 96 | 16.7 ^a^ |
|  |  | *in vivo* |  | NA | NA | NA | NA |  | 28 | 328 | 167 | 50.9 ^b^ |  | 50 | 576 | 55 | 9.5 ^b^ |
| Bovine Taurus alpha satellite I (BTαS) | Prepubertal | cAMP30 |  | 29 | 256 | 168 | 65.6 |  | 26 | 222 | 140 | 63.1 ^abc^ |  | 45 | 391 | 147 | 37.6 ^c^ |
|  |  | DMSO30 |  | 30 | 264 | 164 | 62.1 |  | 33 | 287 | 151 | 52.6 ^c^ |  | 36 | 312 | 115 | 36.9 ^bc^ |
|  |  | TCM24 |  | 27 | 235 | 167 | 71.1 |  | 28 | 249 | 153 | 61.4 ^abc^ |  | 34 | 301 | 48 | 15.9 ^a^ |
|  | Adult | cAMP30 |  | 26 | 225 | 162 | 72.0 |  | 29 | 251 | 177 | 70.5 ^a^ |  | 46 | 405 | 111 | 27.4 ^b^ |
|  |  | DMSO30 |  | 37 | 322 | 213 | 66.1 |  | 31 | 276 | 179 | 64.9 ^abc^ |  | 45 | 394 | 66 | 16.8 ^a^ |
|  |  | TCM24 |  | 29 | 248 | 152 | 61.3 |  | 29 | 254 | 173 | 68.1 ^ab^ |  | 47 | 415 | 152 | 36.6 ^bc^ |
|  |  | *in vivo* |  | NA | NA | NA | NA |  | 30 | 249 | 141 | 56.6 ^bc^ |  | 47 | 411 | 120 | 29.2 ^bc^ |

Values with different superscripts differ significantly for the respective satellite and developmental status(a, b, c).
