# Supplementary material for: Cyclic AMP Affects Oocyte Maturation and Embryo Development in Prepubertal and Adult Cattle
Source: PLoS One. 2016 Feb 29;11(2):e0150264. doi: 10.1371/journal.pone.0150264 (PMC4771806; doi:10.1371/journal.pone.0150264)
Supplement: S5 Table — (DOCX) [file pone.0150264.s010.docx]

S5 Table. Blastocyst cell numbers in expanded blastocysts derived from adult and prepubescent oocytes treated pre and during IVM with cAMP modulators.

| Donors | Treatment | Total evaluated blastocysts | Total cells | ICM | TE | ICM/Total cells (%) |
| --- | --- | --- | --- | --- | --- | --- |
| Prepubertal | cAMP30 | 6 | 152.8±12.9 | 38.7±4.9 | 114.2±9.4 | 25.1±2.0 |
|  | DMSO30 | 6 | 134.0±8.5 | 32.0±6.8 | 102.0±8.1 | 23.7±4.3 |
|  | TCM24 | 6 | 164.3±5.3 | 38.8±3.8 | 125.5±7.4 | 23.9±2.7 |
| Adult | cAMP30 | 6 | 157.5±11.8 | 44.7±5.2 | 112.8±7.8 | 28.1±2.0 |
|  | DMSO30 | 6 | 162.8±9.3 | 34.2±5.4 | 128.7±11.3 | 21.5±4.2 |
|  | TCM24 | 6 | 144.5±7.1 | 32.0±3.0 | 112.5±8.1 | 22.5±2.7 |
|  | *In vivo* | 6 | 155.3±6.8 | 44.3±4.8 | 111.0±6.1 | 28.5±2.6 |

Data are the mean ± SEM. Inner cell mass (ICM), trophectoderm (TE). Data are presented descriptively.
